# Reliability of isokinetic tests of velocity‐ and contraction intensity‐dependent plantar flexor mechanical properties

**DOI:** 10.1111/sms.13920

**Published:** 2021-03-23

**Authors:** Matheus D. Pinto, Cody J. Wilson, Anthony D. Kay, Anthony J. Blazevich

**Affiliations:** ^1^ Centre for Exercise and Sports Science Research (CESSR) School of Medical and Health Sciences Edith Cowan University Perth WA Australia; ^2^ Centre for Physical Activity and Life Sciences The University of Northampton Northampton UK

**Keywords:** flexibility, muscle stretching, passive and active stretching, range of motion, stiffness

## Abstract

“Flexibility” tests are traditionally performed voluntarily relaxed by rotating a joint slowly; however, functional activities are performed rapidly with voluntary/reflexive muscle activity. Here, we describe the reliabilities and differences in maximum ankle range of motion (ROM_max_) and plantar flexor mechanical properties at several velocities and levels of voluntary force from a new test protocol on a commercially available dynamometer. Fifteen participants had their ankle joint dorsiflexed at 5, 30, and 60° s^−1^ in two conditions: voluntarily relaxed and while producing 40% and 60% of maximal eccentric torque. Commonly reported variables describing ROM_max_ and resistance to stretch were subsequently calculated from torque and angle data. Absolute (coefficient of variation (CV%) and typical error) and relative (ICC_2,1_) reliabilities were determined across two testing days (≥72 h). ROM_max_ relative reliability was good in voluntarily relaxed tests at 30 and 60° s^−1^ and moderate at 5° s^−1^, despite CVs ≤ 10% for all velocities. Tests performed with voluntary muscle activity were only reliable when performed at 5° s^−1^, and ROM_max_ reliability was moderate and CV ≤ 8%. For most variables, the rank order of participants differed between the slow‐velocity, relaxed test, and those performed at faster speeds or with voluntary activation, indicating different information. A person's flexibility status during voluntarily relaxed fast or active stretches tended to differ from their status in the traditional voluntarily relaxed, slow‐velocity test. Thus, “flexibility” tests should be completed under conditions of different stretch velocity and levels of muscle force production, and clinicians and researchers should consider the slightly larger between‐day variability from slow‐velocity voluntarily relaxed tests.

## 1 INTRODUCTION

Maximal joint range of motion (ROM_max_) and resistance to tissue elongation are important physical characteristics that influence an individual's ability to perform movements of daily living or sporting tasks.^1^, ^2^ Additionally, these characteristics are sometimes also associated with muscle strain injury risk.^3^, ^4^ From a functional perspective, a lesser antagonist muscle resistance resulting from a high muscle compliance should allow for the performance of agonist muscle actions at lower energetic/metabolic cost compared to cases in which the antagonist is stiffer.^2^, ^5^ Given the functional importance of both ROM and resistance to tissue elongation as well as the apparent changes that occur with aging,^6^, ^7^ disease,^8^, ^9^ and disuse^10^, ^11^ as well as muscle stretching and strength training interventions,^10^, ^12^, ^13^, ^14^ it is not surprising that tests of both ROM_max_ and resistance to tissue elongation are included in clinical and applied settings.

In research (and some clinical situations), flexibility tests are commonly performed using isokinetic dynamometers to rotate a joint at a low velocity, e.g. ≤5° s^−1^, and without voluntary activation of the muscle (although reflexive activity may be present) in order to determine ROM_max_ and to quantify passive musculo‐articular complex (MAC) mechanical properties.^15^ The slow movement velocity used in the test is justified under the assumptions that (a) tonic/stretch reflexes are not evoked and therefore do not influence ROM_max_ and/or the passive elastic properties of the MAC, and (b) results are not likely to be influenced by viscous properties of the muscle or tendon, which are strain rate‐dependent.^14^, ^15^, ^16^, ^17^, ^18^, ^19^ From a mechanistic perspective, this would allow discrimination between several neural and non‐neural mechanisms underpinning ROM assessment.^20^ Nonetheless, both the slow velocity of the test and the lack of voluntary (and usually reflexive) muscle activity may reduce its functional relevance to activities of daily living or sporting tasks, which are performed at faster joint rotation velocities and usually under the influence of reflexive and/or voluntary muscle activity.^21^, ^22^ Such tests would also be important from a clinical viewpoint, particularly for people with neurological conditions (eg, spasticity, contracture) who may exhibit persistent ongoing muscle activity during activities of daily living.

Based on this, the development of a laboratory‐ or clinic‐based set of tests that allows for the assessment of ROM_max_ and MAC mechanical properties at faster joint rotation velocities and higher levels of muscle resistance would allow for greater scrutiny of the relationship between “flexibility” and function in complex human movement tasks, as well as to track the potential changes in flexibility under conditions more similar to those faced during daily or sporting activities. Importantly, such a test battery would have to be developed using commercially available ergometers (eg, isokinetic dynamometers) in order to (a) make the tests feasible for use by a broader range of clinicians and exercise scientists, in addition to researchers, (b) allow better data replication, and (c) minimize the time and budgetary constraints of self‐manufacturing equipment for testing. However, a test battery would only be clinically meaningful if the information given by the higher‐velocity or active muscle tests differs from that provided by the low velocity, passive (ie, standard) tests. That is, in addition to giving different scores of “flexibility,” it would also have to rank individuals differently within a cohort.^23^ This is important because conclusions made from test outcomes are usually based on an individual's score relative to a cohort (sample) or population, and a change in an individual's rank within a cohort or population would affect the conclusions drawn from the test.

Given the above, the main purpose of the present study was to describe, test the between‐day reliability, and show differences in outcomes obtained from a new test battery designed to assess the ankle plantar flexors ROM_max_ and mechanical properties on a commercially available dynamometer at several velocities and levels of voluntary force. The ankle plantar flexors were chosen as the target of the present study because of their frequent use in daily tasks such as walking and running^24^ and because of its frequent use in scientific research (see ref. 14, for review). Specifically, the aims were to (a) test the relative and absolute between‐day (test‐retest) reliabilities of the outcomes of such tests (ie, ROM_max_, peak joint passive moment, MAC stiffness, and passive elastic energy storage) and (b) determine correlations and changes in the cohort‐based ranks between the test outcomes at higher joint rotation velocities and force levels and the standard test (low velocity, relaxed). Nonetheless, as the performance of a test can influence performances in subsequent tests, and the aim of the present study was to determine whether a longer test battery can be conducted, it was important to determine whether maximal force production and tissue resistance to stretch were altered. We therefore examined whether the test battery could be completed without the test movements themselves influencing neuromuscular performance and MAC mechanical properties, thus invalidating its own results. We hypothesized that (a) tests performed with the muscles relaxed at slow and fast velocities would provide “good to excellent” reliability, (b) tests performed with muscles voluntarily active would provide “good” reliability only when performed at the slow stretching velocity, (c) both the relaxed, faster (30≥° s^−1^) and the active, slow‐velocity tests would be uncorrelated with, and rank individuals differently within the cohort than, the traditional voluntarily relaxed, slow‐velocity test, and (d) no significant changes in force and muscle activity would be detected after the completion of the test battery. The present results may provide important methodological information for the assessment of variables that describe an individual's flexibility characteristics (ROM_max_ and tissue resistance to stretch) that may be completed under conditions of different stretch velocities and levels of muscle force in clinical, research, and sport settings.

## 2 METHODS

### 2.1 Participants

Fifteen active men (age = 27.6 ± 6.9 y, mass = 78.3 ± 11.8 kg, height = 1.76 ± 0.06 m) free from neuromuscular disease or musculoskeletal injuries and a minimum 20° dorsiflexion ROM_max_ during a slow‐velocity ankle stretch (ie, 5° s^−1^; knee fully extended) volunteered for the present study. Before participation, participants read and signed an informed consent form, and all participants completed a pre‐exercise medical screening questionnaire to identify any cardiovascular, neurological, or musculoskeletal disease, or any current injury and/or illness that would preclude them from performing maximal effort or passive and active stretching tests. Participants refrained from intense exercise within 48 h of testing and avoided the intake of caffeine or alcohol 6 h prior to the testing sessions. This study was approved by the institutional research ethical committee (project no 19 683).

### 2.2 Overview

**Figure 1:**
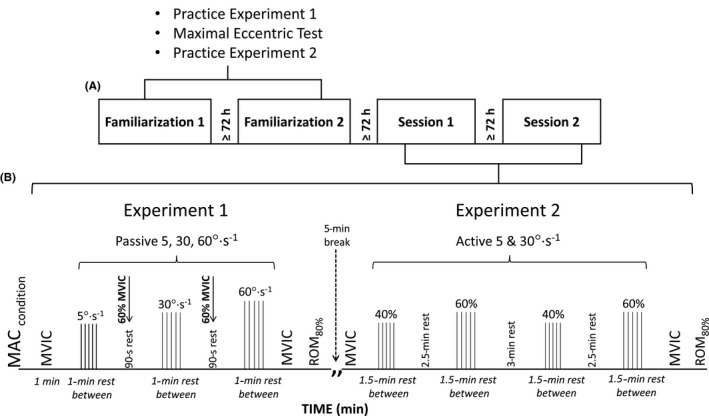
Experimental design. (A) Participants attended the laboratory on four occasions separated by ≥ 72 h. The first and second visits were devoted to familiarization of the test procedures, and the experimental protocols were performed on the third and fourth visits for reliability purposes. In the familiarization sessions, participants were fully familiarized with the maximum range of motion (ROM_max_) passive tests at all joint rotation velocities (for Experiment 1) and with the active ROM_max_ tests (for Experiment 2). Before practicing tests for Experiment 2, participants performed two to three maximal voluntary eccentric plantar flexion contractions (Ecc‐MVC) to 90% of their maximum dorsiflexion joint angle achieved in the passive tests to determine the contraction intensity to be used during the active ROM_max_ tests in Experiment 2. (B) Experimental design, showing the timeline for the performance of maximal voluntary isometric contractions (MVICs) and passive (Experiment 1), performed at 5, 30 and 60° s^−1^, and active (Experiment 2) ROM_max_ stretching tests that were performed at 5 and 30° s^−1^. 5 min of passive rest separated Experiments 1 and 2. MAC condition: plantar flexor isometric contractions at increasing intensities to condition the musculo‐articular complex; ROM_80%_: passive dorsiflexion at 5° s^−1^ to 80% of ROM_max_ achieved in the low‐velocity stretching test; 60% MVIC: 5‐s sub‐maximal conditioning contraction at 60% of MVIC

Participants visited the laboratory on four occasions each separated by ≥ 72 h. The first two visits were devoted to extensive familiarization of the test procedures, while the experimental protocols for two separate experiments, in which Experiment 1 (relaxed muscle stretches) was always performed before Experiment 2 (active muscle stretches), were performed on the third visit (Session 1) and then repeated on the fourth visit (Session 2) to assess inter‐day reliability (see Figure 1A). Experiment 1 was performed before Experiment 2 since moderate volumes of passive stretching are unlikely to induce muscle damage and influence tendon properties whereas active muscle contractions present a greater risk.^25^ The experimental sessions were separated by 3‐13 days except for one individual. Data were analyzed both with and without this individual, although the overall results were not meaningfully affected (see Appendix S1 for analyses completed with the participant removed). Tests were conducted at approximately the same time of the day ± 2h.

### 2.3 Familiarization Sessions

During familiarization sessions, participants practiced both sub‐maximal and maximal voluntary isometric (MVIC) and eccentric (Ecc‐MVC) plantar flexor contractions as well as maximal joint range of motion (ROM_max_) tests with the lever arm of an isokinetic dynamometer (Biodex System 4, Biodex Medical Systems, Shirley, New York) rotating the ankle toward dorsiflexion at 5, 30, and 60° s^−1^ while seated with the knee fully extended. Participants performed several ROM_max_ trials at each velocity with the muscles as relaxed as possible in order to become fully familiar with the tests, and in successive attempts were instructed to stop the test (ie, cease the stretch) at 20–30, 50, 80, 90–100, and then 100% of their perceived ROM_max_ in order to gain confidence with the test system. The participants continued familiarization until they expressed confidence with the tests, and no further familiarization was given in the experimental sessions. After these familiarization sessions, it became clear that reliable ROM_max_ tests at 60° s^−1^ could not be done with the muscles active because it was not possible to accurately produce the required joint moment, so this test was omitted from the test battery of Experiment 2.

Ecc‐MVC tests were performed to allow target joint moments to be calculated for use during the active stretches in Experiment 2 (described in detail below). The system range of motion was set from 20° plantar flexion to 90% of maximum dorsiflexion angle obtained during the passive stretches performed at 5 and 30° s^−1^, respectively. From the joint moment‐angle data, target joint moment feedback of 40 and 60% of Ecc‐MVC was calculated for use on the subsequent experimental days (Sessions 1 and 2, described below). The participants also extensively practiced the active stretching trials during the familiarization sessions in order to become familiar with maintaining contraction intensities equivalent to 40% and then 60% of Ecc‐MVC throughout the stretching test and stopping the dynamometer at their perceived ROM_max_.

### 2.4 Experiment 1

Before commencing Experiment 1 in Sessions 1 and 2, five isometric voluntary plantar flexor contractions were performed at increasing intensities of 20, 40, 60, 80, and 100% of perceived maximal effort (30‐s rest between contractions) while seated in the isokinetic dynamometer (knee fully extended, ankle in the anatomical position) to pre‐condition the musculo‐articular complex (MAC) for subsequent strain.^26^ Two MVICs with a 1‐min rest interval were then performed to obtain maximum isometric peak moment and the maximum level of plantar flexor muscle activity (EMG; see below). This test was repeated at regular intervals throughout the protocol (described below) and the data used to (a) normalize EMG data obtained during muscle stretches, (b) test whether changes in neuromuscular performance were induced by completion of the test battery, and (c) set the isometric contraction target of 60% MVIC that was later performed between ROM_max_ tests in Experiment 1.

Subsequently, the participants completed Experiment 1, which included ROM_max_ tests with the lever arm of the dynamometer rotating the ankle toward dorsiflexion at 5, 30, and 60° s^−1^, with 2‐5 trials being performed (criteria relating to the number of trials are detailed below) and with the participants keeping their muscles as relaxed as possible. Sixty seconds of rest was given between stretches of the same velocity, and 90 s was given between stretch velocities. Angular velocities were always presented from slowest to fastest because the rate of decrease in stiffness across repeated stretches has been reported to be greater when fast stretching angular velocities are imposed.^16^ Within the 90‐s rest periods between‐test velocities, participants performed a 5‐s sub‐maximal conditioning contraction at 60% of MVIC to test for numbness (Figure 1).

For the stretches, the participants were positioned on the chair of an isokinetic dynamometer with the hip angle at 55° (ie, semi‐reclined), knee fully extended (0°), the ankle in the anatomical position (0°; sole of the foot perpendicular to the shank), and the lateral malleolus aligned to the dynamometer's axis of rotation.^25^ A rigid clip strap was tightened across the foot to minimize heel displacement from the dynamometer footplate, which was visually confirmed by the investigators of the study prior to warm‐up. The participant was seated with knee angle ~ 30° flexion before the knee was extended to 0° to take up slack from the dynamometer system.^27^ Thereafter, the participant's ankle was rotated into dorsiflexion from 20° of plantar flexion to full volitional dorsiflexion ROM, defined as the point of discomfort at which the participant could no longer tolerate further stretch, with the stretch terminated when the participant pressed a dynamometer control button. Maximal dorsiflexion range of motion was calculated from anatomical position (0° dorsiflexion). For all tests, visual feedback of foot rotation was blocked using a cover placed over the thigh. Participants were asked to completely relax their muscles while muscle activity (EMG) feedback was given instantaneously on a screen placed in front of them. Upon completion of the experiment, the participants performed plantar flexor MVICs and then had their ankle rotated into dorsiflexion at a slow velocity (5° s^−1^) to 80% of ROM_max_ achieved in the low‐velocity joint rotation test (Figure 1B) in order to confirm whether the repeated stretch attempts might have induced changes in neuromuscular performance and/or MAC mechanical properties; the 80% ROM_max_ intensity was chosen in order to impose substantial stress on the MAC but without adding to the number of maximal stretches performed. Their foot was then released from the dynamometer, and they were allowed to stand.

### 2.5 Experiment 2

Experiment 2 commenced 5 min after completion of Experiment 1 in Sessions 1 and 2 after participants were re‐positioned in the isokinetic dynamometer as per Experiment 1. Active ROM_max_ tests were performed at 5 and 30° s^−1^ while maintaining contraction intensities equivalent to 40% (±5 Nm; Ecc‐40) and then 60% (±5 Nm; Ecc‐60) of Ecc‐MVC achieved in the familiarization sessions. These relatively low contraction levels were chosen to minimize the fatigue effects induced by higher contraction levels. Ecc‐40 was always performed before Ecc‐60.

**Figure 2:**
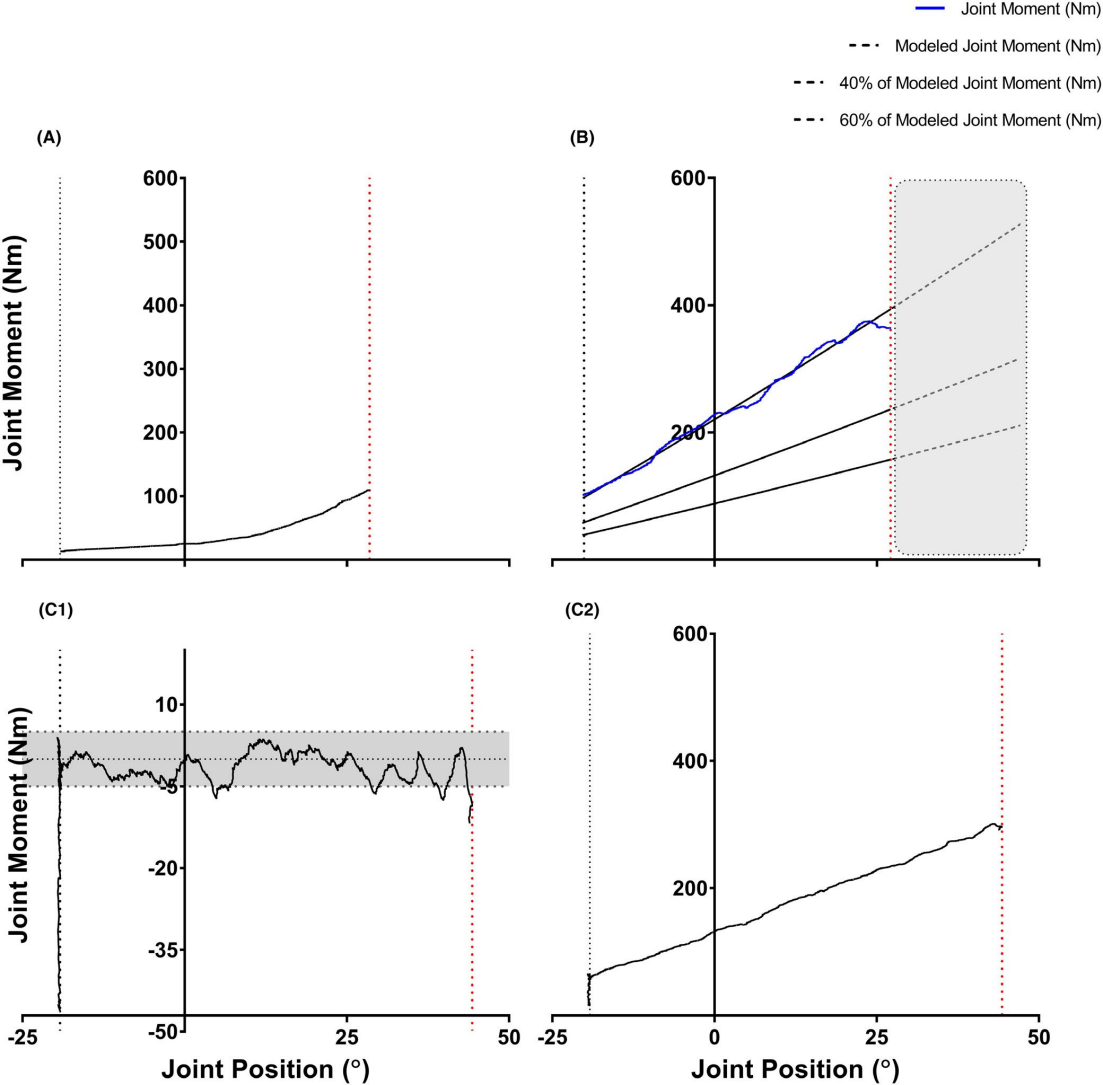
Schematic representation of the procedures used to determine the target joint moment levels during active stretching tests, and the visual feedback provided, using data from one subject. The joint angle (from neutral) corresponding to 90% of the maximal range of motion (ROM_max_) during the passive, 5° s^−1^ joint rotation was calculated and then used during the maximal plantar flexion eccentric contractions at 5 and 30° s^−1^ (Ecc‐MVC; Panel A). A second‐ or third‐order polynomial regression line was fitted to the best Ecc‐MVC moment‐angle data from contraction start to 95% of the final joint angle of Ecc‐MVC (blue line; Panel B). The resulting regression equation enabled estimation of joint moments at angles greater than those used in the Ecc‐MVC test (shaded area in Panel B) and calculation 40% and 60% of the maximal moment at each joint angle in order to provide target lines for subsequent eccentric contractions, that is, “active” stretches at 40% (Ecc‐40) and 60% (Ecc‐60) of Ecc‐MVC. The difference between the active moment and target contraction intensity should equal to zero in order to maintain the contraction intensity required, and this difference was visually provided in real time so that the participants were able to maintain and adjust the target plantar flexion moment within ± 5 Nm during the active stretch protocol (shaded area in Panel C1). Note that, for this participant, ±5 Nm was approximately 1%‐9% of the 60‐Ecc joint moment developed during the test, depending on the joint angle calculated. Panels C1 and C2 show the visual feedback (C1) provided to participants during the active maximum range of motion stretching trial as well as the actual joint moment‐angle curve (C2) during the tests (5° s^−1^; 60% Ecc‐MVC). The vertical black dashed line represents the start of stretch, whereas the vertical red dashed lines represent joint positions at end of stretch (maximum range of motion except for panel B that final ROM was pre‐set)

To set the on‐screen target line for the tests, second‐ or third‐order polynomial regression lines were fitted to the Ecc‐MVC moment‐angle data (best trial collected in familiarization 1 or 2) from contraction start to 95% of the final joint angle of Ecc‐MVC; the full data range was not used because some joint moment fluctuation within the final range was noticed during pilot tests that considerably affected the models. The resulting regression equations enabled (a) estimation of joint moments at angles greater than those used in the Ecc‐MVC test, and (b) calculation of 40% and 60% of the maximal moment at each joint angle in order to provide target lines for subsequent eccentric contractions, that is, “active” stretches at 40% (Ecc‐40) and 60% (Ecc‐60) of Ecc‐MVC. Because the software only allowed a constant signal to be used as feedback, separate channels were created to show a varying feedback signal using the difference between the active moment (Figure 2, Panel C2) and the target contraction intensity (given by the polynomial equation calculated). When the difference between these two signals equaled zero the participant was known to be maintaining the required contraction intensity. This difference was provided visually in real time (with ±5 Nm boundary lines) during tests so that the participants were able to maintain and adjust the target plantar flexion moment within ± 5 Nm during the active stretch trials (Figure 2, shaded area in Panel C1). Figure 2 provides an example of the calculation process. The participants were instructed to maintain the joint moment level between the guidelines throughout the ROM (see Figure 2, Panel C1), which was volitionally terminated by pressing a hand held button at a “point where they could no longer tolerate being stretched” or when they could not self‐adjust their voluntary joint moment within the guidelines, that is, target joint moment. This was verbally acknowledged by participants upon stretching test termination. Stretches were performed at two angular velocities (5 and 30° s^−1^) with 90‐s rest intervals between trials, 150 s between stretches with different contraction intensities, and 180 s between stretches at different test velocities. Upon completion of Experiment 2, the participants again performed plantar flexor MVICs and then had their ankle rotated into dorsiflexion at a slow velocity (5° s^−1^) to 80% of ROM_max_ achieved in the low‐velocity joint rotation test (Figure 1B) in order to confirm whether the test protocol might have induced changes in neuromuscular performance and/or MAC mechanical properties.

Experiments 1 and 2 were repeated under similar experimental conditions on a separate day (Session 2) in order to test passive and active ROM_max_ for between‐day reliability. No significant differences were observed between the total number of stretching trials performed within each experiment between sessions (*P* > 0.60).

### 2.6 Number of stretching trials: *a‐priori* criteria

Two stretch trials were always provided in each condition in both Experiments 1 and 2, with additional trials performed if a difference ≥ 5% of ROM_max_ was observed. In the unusual event that a participant did not meet this criterion, a 2.5° difference between trials was deemed acceptable but the maximum number of trials was set at 5. These criteria were set *a priori* and were met by all participants with the exception of one participant in Experiment 2 of Session 1 who completed the Ecc‐60 tests but did not achieve the criterion for inclusion; for this participant, a 3° difference was observed in the two trials with least difference.

### 2.7 Selection of trials for analysis (*post hoc*)

In Experiment 1, inspection of the joint moment‐angle of all tests was performed *post hoc* to select the trial for analysis. The two trials with least ROM_max_ difference, and the trial with the greatest ROM_max_ if not within them, were inspected for variability of the joint moment‐angle relation. When the greatest ROM_max_ presented abnormal variations in the joint moment‐angle relation or it significantly differed from the two trials with least ROM_max_ difference, the best of the two trials with least difference in ROM_max_ was selected for analysis. The within‐day percent and absolute mean differences between the two most similar trials in Session 1 were 3.3 ± 2.7% and 1.2 ± 0.9°, 2.1 ± 2.4% and 0.8 ± 1.0°, and 2.9 ± 4.0% and 1.2 ± 1.6°, for stretching tests performed at 5, 30, and 60° s^−1^, respectively. Similarly, percentage and absolute differences between these trials in Session 2 were 2.3 ± 1.7% and 0.8 ± 0.5°, 1.8 ± 2.3% and 0.7 ± 0.8°, and 1.6 ± 1.8% and 0.7 ± 0.7°, for stretching tests performed at 5, 30, and 60° s^−1^, respectively.

In Experiment 2, joint moments sometimes fluctuated outside of the target. Therefore, mean absolute and root mean square errors from the target were calculated and correlated with ROM_max_ to determine whether these fluctuations were associated with a greater or lesser ROM_max_ result. Trials with greatest and least ROM_max_ were randomly selected between Sessions 1 and 2, and errors were calculated throughout the stretching trial and in the last 10° of ROM (the range with greatest deviations from target and most likely to influence ROM_max_). A statistical relationship was not observed using Pearson's product‐moment correlation analyses between deviations from target and ROM_max_ for Ecc‐40 and Ecc‐60 stretching tests (*P* > 0.1), indicating that a participant's ability to maintain the joint moment within the target was not associated with the ROM_max_ achieved during the ROM_max_ test (see Appendix S2 for detailed results). Inspection of the plantar flexor EMG signal within the last degrees of range of motion confirmed that EMG was greater than baseline (resting EMG) for all participants; that is, muscles were active through the full range in these trials. As in Experiment 1, the two trials with least ROM_max_ difference, and the trial with the greatest ROM_max_ if not within them, were inspected for variability of the joint moment‐angle relation. When the greatest ROM_max_ presented abnormal variations in the joint moment‐angle relation or it significantly differed from the two trials with least ROM_max_ difference, the best of the two trials with least difference in ROM_max_ was selected for analysis. The within‐day percent and absolute mean differences between the two most similar trials in Session 1 were 3.1 ± 3.1% and 1.1 ± 1.2° and 2.6 ± 2.2% and 1.0 ± 0.8°, for stretching tests performed at 5° s^−1^ with contraction intensities of Ecc‐40 and Ecc‐60, respectively. Percentage and absolute differences between trials in Session 2 were 2.9 ± 1.7% and 1.1 ± 0.7° and 2.9 ± 2.0% and 1.1 ± 0.8°, for stretching tests performed at 5° s^−1^ with contraction intensities of Ecc‐40 and Ecc‐60‐Ecc, respectively.

### 2.8 Joint position, moment, angular velocity, and angular acceleration data collection (Experiments 1 and 2)

During each trial, passive joint moment, joint position, and joint angular velocity data were recorded from the dynamometer, and joint acceleration was subsequently derived from the velocity‐time data. The start of stretch was determined *post hoc* as the last signal deflection that was greater or equal to two standard deviations of the average baseline unfiltered velocity. ROM_max_ was defined as the joint angle at the point where angular acceleration became negative (ie, footplate deceleration) and the acceleration signal crossed zero and did not return to baseline at the end of the constant‐velocity phase of the stretch.^28^ This point was assumed to indicate the point at which the participant pushed the button to volitionally stop the stretch and occurs slightly before the maximum angle achieved by the ankle.

### 2.9 Peak passive and active joint moment, musculo‐articular complex (MAC) stiffness, and passive and active elastic energy storage calculation (Experiments 1 and 2)

Passive and active ROM_max_ trials enabled ROM_max_, peak passive and active joint moments (stretch tolerance), passive and active joint moment‐angle relation gradients (MAC stiffness calculated in several ranges of the joint moment‐angle relations), and areas under the passive and active joint moment‐angle relations (elastic potential energy storage) to be calculated. These variables were calculated because they are common variables calculated in previous studies^15^ and cover a broad spectrum of joint ROMs achieved in sporting tasks and activities of daily living.^1^, ^2^ Peak passive and active moments were calculated as the moment at ROM_max_, whereas passive and active elastic energies were calculated as the areas under the passive and active moment‐angle relations, respectively, from anatomical position to ROM_max_ (J). Gradients of the passive and active moment‐angle relations were calculated as the ankle moment change per joint angle change (i) from neutral to 10° and 20° of dorsiflexion, (ii) through the last 10° of dorsiflexion, and (iii) from 0° to ROM_max_.

### 2.10 Muscle activity during stretch and MVIC (EMG) (Experiments 1 and 2)

During MVICs and both passive and active stretches, the surface electromyogram (EMG) signal was recorded from gastrocnemius medialis (EMG_GM_), soleus (EMG_Sol_), gastrocnemius lateralis (EMG_GL_), and tibialis anterior (EMG_TA_) using bipolar configurations of two Ag/AgCl self‐adhesive electrodes (inter‐electrode distance of 20 mm; Blue Sensor N‐00‐S, 28mm^2^, Ambu). After appropriate skin preparation, electrodes were positioned along the expected line of the fascicles and in accordance with Surface EMG for Non‐Invasive Assessment of Muscles (SENIAM) guidelines, and the reference electrode was placed on the lateral malleolus. EMG signals were recorded synchronously with joint moment, joint angle, joint angular velocity data at a 2,000‐Hz analogue‐digital conversion rate, and band‐pass filtered at 20–400 Hz (BioAmp EMG System, ADInstruments; gain = 1000, input impedance = 200 MΩ, common mode rejection ratio ≥ 85 dB at 1–60 Hz, noise input 1.3 μV RMS).

EMG data were smoothed in real time using a symmetric root mean square (RMS) filter with a 100‐ms averaging window. Maximum EMG_GM_, EMG_GL_, EMG_Sol_, and EMG_TA_ amplitudes were collected from a 2‐s epoch around the peak moment during MVIC (best trial) and were averaged as a representation of the total plantar flexor muscle activity. During passive ROM_max_ tests in Experiment 1, EMG activities were recorded from all four muscles and provided in real time to participants to help them maintain a low muscle activity during stretches. Although participants were asked to fully relax their muscles, some level of EMG was always present at some point within a trial. However, as the participants were well familiarized with the stretching and made a conscious effort to remain relaxed during the tests, all trials were considered for analysis. The level of muscle activity was also recorded, but not shown to participants, during the active stretches in Experiment 2. Additionally, EMG signals were collected with the participant fully relaxed and resting quietly before commencing the MVIC. The mean RMS EMG activity within a 3‐s epoch was calculated, and this background noise was subtracted from the RMS EMG recorded from all trials.

### 2.11 Maximal isometric joint moment, sub‐maximal range of motion, and EMG amplitude calculations (Experiments 1 and 2)

In order to determine whether stretching tests performed early in an experiment influenced neuromuscular function or tissue mechanical properties in later tests, MVICs (0° joint angle) and passive stretching tests (5° s^−1^, to 80% of ROM_max_) were performed before and after completion of the ROM_max_ tests in Experiments 1 and 2. The peak isometric moment and total plantar flexion EMG (average of EMG from all muscles) were measured during a 2‐s epoch at the plateau of the moment‐time trace during the first MVIC trial. The first trial was examined because only one MVIC attempt was allowed after the stretching tests and no prior muscle conditioning was performed. In addition, the passive joint moment at 80% of ROM_max_, total plantar flexion (normalized to the best MVIC) and tibialis anterior (co‐activation) EMG within the last 2°, and MAC stiffness in the last 10° of the joint rotation were calculated from the stretch test data.

### 2.12 Statistical analysis

Relative and absolute between‐day (test‐retest) reliabilities were calculated for all variables using intra‐class correlation coefficients (ICCs) and their respective 95% confidence intervals (CIs). Between‐participant relative reliability was calculated using two‐way mixed‐effect models, absolute agreement (systematic errors) single (non‐averaged) scores (ICC_2,1_). ICC values < 0.5 were considered indicative of poor reliability, values 0.5 ‐ 0.75 indicated moderate reliability, values 0.75 ‐ 0.9 indicated good reliability, and values > 0.9 indicated excellent reliability. Absolute reliability was calculated using the typical error (ie, standard error of measurement, SEM) given by *SD_diff_*/√2 where *SD_diff_* is the standard deviation of the difference in scores from day 2 to day 1.^29^ To determine the minimum difference for a change between trials to be considered “real,” the minimum detectable change (MDC) was calculated as SEM × 1.96^30^ Individual coefficients of variation (CV%) were calculated for each participant as the between‐day standard deviation divided by the between‐day average multiplied by 100, and then, individuals’ CVs were averaged. While some researchers arbitrarily consider CVs ≤ 10%‐15% as “good” reliability, ^31^ in some cases these CVs may be considered “low.” Such an arbitrary decision may lead to incorrect conclusions of test reliability, and therefore, we have decided not to adopt such criteria in the present study.

Descriptive data are shown as mean ± standard deviation (mean ± SD), and the normality of all values was verified by the Shapiro‐Wilk test. Data without normal distribution were log‐transformed before parametric analysis. When log‐transformation did not result in parametric data, Wilcoxon signed‐rank tests were used. Paired‐samples’ *t* tests and Wilcoxon signed‐rank tests were used to explore systematic differences between days. In addition, paired‐samples’ *t* tests were used to test whether the performance of ROM_max_ tests in Experiments 1 and 2 affected MVIC joint moment and muscle activity (stretch‐induced force loss), while repeated‐measures ANOVAs were used to test for changes in passive musculo‐articular mechanical properties and reflexive muscle activity (at 80% of ROM_max_) before and after Experiments 1 and 2. Hedges’ standardized effect sizes were calculated as the difference between the score means divided by the pooled standard deviation of the scores.

Spearman's rank‐order correlation coefficients (r*_s_*) were computed to quantify the strength of relationships between outcome variables (ie, ROM_max_, peak moment, MAC stiffness, and elastic energy) from tests performed at 5, 30, and 60° s^−1^ by ranking the scores within the cohort (rank‐order manner). In addition, individual cohort rank scores (ranked against others within the sample, Z scores) of these variables were used to determine whether conclusions differed between tests performed at the three joint rotation angular velocities (Experiment 1) and contraction intensities (Experiment 2). Very strong (≥0.9) r*_s_* and homogeneous linearity in the individual Z scores for each joint rotation velocity (lines not crossing each other in individually plotted Z scores graphs) were deemed to indicate identical test information. All data were analyzed using SPSS statistical software (version 25; SPSS) with a level of significant set *a priori* at α = 0.05.

## 3 RESULTS

### 3.1 Experiment 1 (passive stretches)

#### 3.1.1 Descriptive statistics and systematic differences

Descriptive statistics are reported in Table 1 as mean ± SD. Paired‐samples’ t tests and Wilcoxon signed‐rank tests revealed no significant systematic differences in any dependent variables (*P* > 0.1) for stretching tests performed at 5, 30, and 60° s^−1^, with the exception of MAC stiffness from 0°–ROM_max_ at 30° s^−1^ (0.5 Nm ^−1^ mean difference (8.2 ± 16.4%), ES = 0.4, t = 2.26, *P* = 0.04) and ROM_max_ in stretching tests at 60° s^−1^ (1.5° mean difference (3.6 ± 4.2%), ES = 1.08, Z = −2.613, *P* = 0.009). Note that two outliers were observed in the ROM_max_ analyses for tests performed at 60° s^−1^, and hence, a separate analysis excluding these participants was performed. Paired‐samples’ t tests again revealed a small but statistically greater ROM_max_ (1.4° mean difference [3.1 ± 4.3%]) in the second compared to the first experimental session (ES = 0.89, t = −2.53, *P* = 0.026; n = 13).

**Table 1:** Descriptive statistics for maximum dorsiflexion angle, peak passive joint moment, musculo‐articular (MAC) stiffness, and passive elastic energy from passive stretching tests performed at 5, 30, and 60° s^−1^ obtained in Sessions 1 and 2 (ie, 3rd and 4th visits)

	Passive ankle joint rotation velocities					
	5° s^−1^		30° s^−1^		60° s^−1^	
	Session 1	Session 2	Session 1	Session 2	Session 1	Session 2
Maximum dorsiflexion angle (°)	34.8 ± 6.3	36.5 ± 6.2	42.0 ± 4.04	43.4 ± 4.6	40.4 ± 4.6	41.9 ± 4.43*
Peak passive moment (Nm)	158.9 ± 63.3	159.7 ± 56.8	248.3 ± 64.9	237.6 ± 73.2	257.0 ± 73.0	258.0 ± 91.9
Passive elastic energy (J)	49.5 ± 23.8	52.3 ± 23.0	96.1 ± 33.9	92.2 ± 39.2	103.2 ± 38.4	103.5 ± 48.6
MAC stiffness (Nm °^−1^)						
0–10°	1.32 ± 0.61	1.37 ± 0.65	2.57 ± 1.26	2.23 ± 1.34	3.4 ± 1.39	3.20 ± 1.76
0–20°	1.74 ± 0.76	1.75 ± 0.76	3.05 ± 1.22	2.73 ± 1.37	4.0 ± 1.43	3.81 ± 1.92
Last 10°	6.05 ± 2.37	5.81 ± 2.05	6.76 ± 1.58	6.73 ± 2.42	6.19 ± 1.20	5.93 ± 1.96
0° – ROM_max_	3.57 ± 1.34	3.37 ± 1.18	5.08 ± 1.29	4.57 ± 1.18*	5.27 ± 1.38	5.25 ± 1.88

* Statistically different from Day 1, *P* < 0.05.

#### 3.1.2 Relative and absolute reliability

Relative (ICC_2,1_) and absolute (typical error, MDC, and CV%) between‐ and within‐participant test‐retest reliability statistics for all variables are presented in Table 2.

**Table 2:** Relative and absolute (test‐retest) reliability statistics for passive stretches. Intra‐class correlation coefficient (ICC_2,1_) indicates relative reliability while standard error of measurements (SEM, ie, typical error), coefficients of variation (CV%), and minimal detectable changes (MDC) indicate absolute reliability

	Passive ankle joint rotation velocities											
	5° s^−1^				30° s^−1^				60° s^−1^			
	ICC (95% CI)	SEM	CV (%)	MDC	ICC (95% CI)	SEM	CV (%)	MDC	ICC (95% CI)	SEM	CV (%)	MDC
Maximum dorsiflexion angle (°)	0.54 (0.08 to 0.82)	4.20	9.97	8.24	0.79 (0.46 to 0.93)	1.83	3.75	3.59	0.87 (0.48 to 0.96)	1.30	3.35	2.54
Peak passive joint moment (Nm)	0.68 (0.27 to 0.88)	34.6	17.0	67.8	0.75 (0.41 to 0.91)	34.7	12.2	68.0	0.82 (0.55 to 0.94)	35.8	9.77	70.1
Passive elastic energy (J)	0.70 (0.30 to 0.89)	13.1	20.9	25.7	0.85 (0.61 to 0.95)	14.5	14.9	28.3	0.86 (0.63 to 0.95)	17.0	14.4	33.2
MAC stiffness (Nm·°^−1^)												
0–10°	0.78 (0.45 to 0.92)	0.31	18.5	0.60	0.85 (0.59 to 0.95)	0.46	23.1	0.90	0.74 (0.39 to 0.91)	0.81	22.6	1.59
0–20°	0.80 (0.49 to 0.93)	0.35	18.8	0.69	0.77 (0.46 to 0.92)	0.60	22.1	1.18	0.79 (0.48 to 0.92)	0.79	17.8	1.56
Last 10°	0.57 (0.09 to 0.83)	1.48	21.9	2.89	0.64 (0.20 to 0.86)	1.26	15.2	2.47	0.71 (0.33 to 0.89)	0.88	14.2	1.73
0° – ROM_max_	0.70 (0.33 to 0.89)	0.69	17.0	1.35	0.71 (0.30 to 0.89)	0.61	11.0	1.20	0.75 (0.40 to 0.91)	0.84	10.8	1.65

### 3.2 Associations between variables in passive tests (Experiment 1) at different velocities

#### 3.2.1 Session 1

As shown in Table 3, Spearman's rank‐order correlations between 5 and both 30 and 60° s^−1^ were moderate‐to‐strong in ROM_max_, peak passive joint moments, passive elastic energy, and MAC stiffness calculated from neutral to 10° and 20° of dorsiflexion, and from 0° to ROM_max_. However, no significant correlations were observed for MAC stiffness calculated through the last 10°, or for MAC stiffness calculated from neutral to 10° and 20° of dorsiflexion between 5 and 30° s^−1^. On average, individuals ranked differently within the cohort in tests at each velocity, so their rank in a test at one velocity may be dissimilar to their rank in a test performed at different velocity (see Appendix S3 for detailed results and graphs).

**Table 3:** Associations between variables obtained in passive stretching tests performed at different velocities

	Passive ankle joint rotation velocities											
	5 vs. 30° s^−1^						5 vs. 60° s^−1^					
	r_s_ (95% CI)		Average change in rank		% change in rank		r_s_ (95% CI)		Absolute change in rank		% change in rank	
	Session 1	Session 2	Session 1	Session 2	Session 1	Session 2	Session 1	Session 2	Session 1	Session 2	Session 1	Session 2
Maximum dorsiflexion angle (°)	0.59 (0.10 to 0.80)*	0.52 (0.0007 to 0.82)*	3.3	3.1	22.2	20.4	0.60 (0.1 to 0.8)*	0.50 (−0.05 to 0.80)	3.5	3.2	23.1	21.3
Peak passive joint moment (Nm)	0.78 (0.40 to 0.90)**	0.67 (0.22 to 0.90)**	2.3	2.5	15.1	16.0	0.78 (0.4 to 0.9)**	0.57 (0.06 to 0.84)*	2.3	2.8	15.1	18.7
Passive elastic energy (J)	0.66 (0.20 to 0.90)**	0.64 (0.18 to 0.87)*	2.9	2.5	19.6	16.9	0.70 (0.3 to 0.9)**	0.59 (0.09 to 0.85)*	2.7	2.8	17.8	18.7
MAC stiffness (Nm °^−1^)												
0–10°	0.50 (−0.03 to 0.81)	0.74 (0.36 to 0.91)**	3.6	2.3	24.0	15.1	0.64 (0.17 to 0.87)*	0.72 (0.31 to 0.90)**	3.1	2.1	20.4	14.2
0–20°	0.47 (−0.07 to 0.80)	0.53 (0.01 to 0.83)*	3.6	3.1	24.0	20.4	0.71 (0.30 to 0.90)**	0.54 (0.03 to 0.83)*	2.7	3.2	17.8	21.3
Last 10°	0.4 (−0.10 to 0.80)	0.60 (0.12 to 0.86)*	3.9	2.7	25.8	17.8	0.4 (−0.10 to 0.80)	0.66 (0.21 to 0.86)**	3.7	3.1	24.9	20.4
0° – ROM_max_	0.9 (0.71 to 0.97)**	0.68 (0.24 to 0.89)**	1.3	2.8	8.9	18.7	0.88 (0.66 to 0.96)**	0.59 (0.09 to 0.85)*	1.5	2.9	9.8	19.6

* Significant to *P* < 0.05;

** Significant to *P* < 0.01.

#### 3.2.2 Session 2

As shown in Table 3, Spearman's rank‐order correlations between 5 and both 30 and 60° s^−1^ were moderate‐to‐strong in ROM_max_, peak passive joint moments, passive elastic energy, and MAC stiffness calculated all ranges, but no correlation was observed for ROM_max_ between 5 and 60° s^−1^. On average, individuals ranked differently within the cohort in tests at each velocity, so their rank in a test at one velocity may be dissimilar to their rank in a test performed at different velocity (see Appendix S3 for detailed results and graphs).

### 3.3 Experiment 2 (active stretches)

#### 3.3.1 Descriptive statistics and systematic differences

Descriptive statistics are reported in Table 4 as mean ± SD. Note that one participant could not self‐adjust the voluntary moment within the guidelines in joint stretching tests at 40‐Ecc because the required joint moment was too low to maintain within the target guidelines. Therefore, the trials for this participant were excluded with analyses conducted on remaining participants’ data (n = 14). Paired‐samples’ t tests and Wilcoxon signed‐rank tests revealed no significant systematic differences in any dependent variables (*P* > 0.1) for 40‐ and 60‐Ecc stretches performed at 5° s^−1^.

**Table 4:** Descriptive statistics for active maximum dorsiflexion angle, peak active joint moment, active musculo‐articular complex (MAC) stiffness, and active elastic energy from active stretching tests performed at 40 and 60% (40‐Ecc, and 60‐Ecc) of maximal plantar flexion eccentric contractions during 5° s^−1^ obtained in Sessions 1 and 2 (ie, 3rd and 4th visits)

	Active stretching tests performed at 5° s^−1^			
	40‐Ecc		60‐Ecc	
	Session 1	Session 2	Session 1	Session 2
Maximum dorsiflexion angle (°)	38.1 ± 5.8	38.1 ± 7.2	39.6 ± 6.6	40.2 ± 6.1
Peak active joint moment (Nm)	195.4 ± 46.6	188.3 ± 37.7	250.7 ± 45.5	252.6 ± 43.4
Active elastic energy (J)	90.4 ± 26.9	88.8 ± 29.0	135.7 ± 39.7	137.4 ± 38.0
Musculo‐articular complex stiffness (Nm·°^−1^)				
0–10°	2.38 ± 0.62	2.0 ± 0.62	3.17 ± 0.95	3.02 ± 0.96
0–20°	2.04 ± 0.63	2.02 ± 0.58	3.00 ± 0.79	2.97 ± 0.80
Last 10°	4.36 ± 2.91	3.86 ± 2.04	2.99 ± 2.01	3.24 ± 2.45
0° – ROM_max_	2.37 ± 1.09	2.34 ± 0.81	2.74 ± 1.05	2.71 ± 0.97

#### 3.3.2 Relative and absolute reliability

Relative (ICC_2,1_) and absolute (typical error, MDC, and CV%) test‐retest reliability for all variables in active stretching tests performed at 5° s^−1^ in Experiment 2 is presented in Table 5. Note that results from active stretching tests performed at 30° s^−1^ are not included because no participant was able to reliably maintain the required target moment level during this stretching test velocity.

**Table 5:** Relative and absolute (test‐retest) reliability statistics for active stretching tests performed at 40‐ and 60‐Ecc. Intra‐class correlation coefficient (ICC_2,1_) indicates relative reliability, while standard error of measurements (SEM, ie, typical error), coefficients of variation (CV%), and minimal detectable changes (MDC) indicate absolute reliability

	Ankle joint moment feedback for stretching tests performed at 5° s^−1^							
	40‐Ecc				60‐Ecc			
	ICC (95% CI)	SEM	CV (%)	MDC	ICC (95% CI)	SEM	CV (%)	MDC
Maximum dorsiflexion angle (°)	0.68 (0.25 to 0.89)	3.76	7.98	7.38	0.71 (0.33 to 0.89)	3.47	6.63	6.80
Peak passive joint moment (Nm)	0.77 (0.43 to 0.92)	20.4	8.25	40.07	0.91 (0.76 to 0.97)	13.38	4.00	26.2
Passive elastic energy (J)	0.80 (0.48 to 0.93)	12.8	11.91	25.14	0.84 (0.58 to 0.94)	16.11	8.51	31.6
Musculo‐articular complex stiffness (Nm·°^−1^)								
0–10°	0.30 (−0.17 to 0.69)	0.51	20.7	0.99	0.43 (−1.0 to 0.77)	0.73	19.7	1.42
0–20°	0.94 (0.83 to 0.98)	0.15	6.49	0.30	0.88 (0.68 to 0.96)	0.29	6.36	0.56
Last 10°	0.64 (0.20 to 0.87)	1.51	26.7	2.97	0.42 (−0.12 to 0.76)	1.73	165.7	3.31
0° – ROM_max_	0.73 (0.33 to 0.90)	0.51	17.0	1.01	0.98 (0.94 to 0.99)	0.15	4.31	0.30

### 3.4 Associations between ROM_max_ obtained in passive versus active stretching tests

#### 3.4.1 Session 1

**Figure 3:**
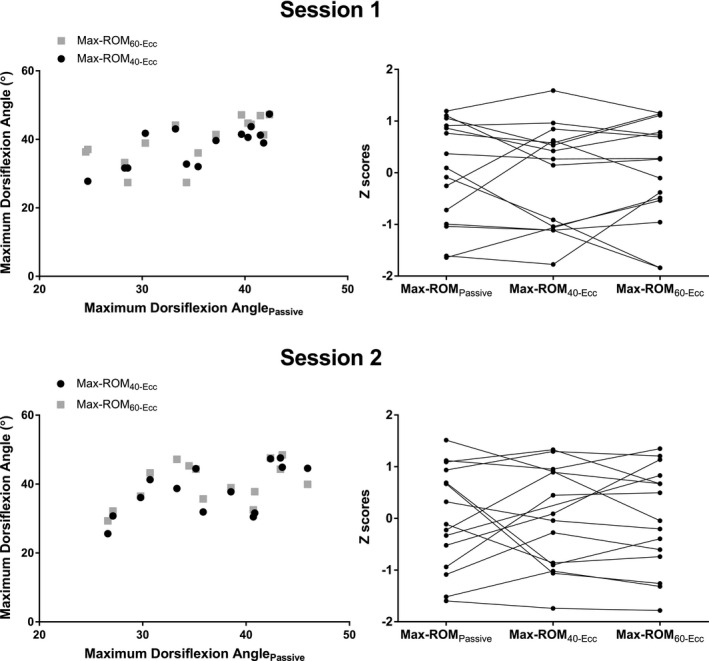
Left Panel: Relationships between maximum joint range of motion (ROM_max_) during 5° s^−1^ passive and active stretches performed at 40‐ and 60‐Ecc from Sessions 1 and 2 (top and bottom, respectively). Overall, moderate‐to‐strong significant relationships were found between ROM_max_ in passive and active tests except for the 60‐Ecc active stretching tests performed in Session 2. Right Panel: The individual relative (to the cohort mean, Z scores) change in scores for ROM_max_ during passive and active stretches. The nonstandard changes in rank scores obtained at each stretching test suggest that different results can be drawn from these tests

Spearman's correlation analysis revealed significant positive moderate‐to‐strong correlations between ROM_max_ obtained in passive and both 40‐Ecc and 60‐Ecc active stretching tests performed at 5° s^−1^ (r*_s_* = 0.61 [95% CI: 0.10 to 0.87], *P* = 0.024 and r*_s_* = 0.72 [95% CI: 0.32 to 0.90], *P* = 0.003). Visual inspection of individual changes revealed no standard pattern of change when rank scores were graphically represented (see Figure 3, right panel). Thus, participants who scored best or worst in passive ROM_max_ did not necessarily maintain this position in active stretching tests at 40‐ and 60‐Ecc. On average, participants were ranked 2.6 (passive vs. active 40‐Ecc) and 2.5 (passive vs. 60‐Ecc) places different within the cohort, equating to 17.1 and 16.9% changes in ranking. The maximum changes in rank between tests were 7 (46.7%) and 6 (40.0%), respectively.

#### 3.4.2 Session 2

Spearman's correlation analysis revealed significant positive moderate‐to‐strong correlations between ROM_max_ obtained in passive and 40‐Ecc active stretching tests performed at 5° s^−1^ (r*_s_* = 0.63 [95% CI: 0.13 to 0.87], *P* = 0.02), but a correlation was not observed in ROM_max_ in passive and 60‐Ecc active stretching tests (r*_s_* = 0.46 [−0.08 to 0.80], *P* = 0.09). Visual inspection of individual changes revealed no standard pattern of change when rank scores were graphically represented (see Figure 3). Thus, participants who scored best or worst in passive ROM_max_ did not necessarily maintain this position in active stretching tests at 40‐ and 60‐Ecc. On average, participants were ranked 2.9 (passive vs. active 40‐Ecc) and 3.7 (passive vs. active 60‐Ecc) places different within the cohort, equating to 19.0 and 24.9% changes in ranking. The maximum changes in rank between tests were 7 (46.6%) and 8 (53.3%), respectively.

### 3.5 Changes in muscle voluntary moment and passive mechanical properties of the musculo‐articular complex

#### 3.5.1 Session 1

In Experiment 1, paired‐samples’ t tests revealed no significant changes in MVIC (1.3 ± 16.9%, t = 0.18, *P* = 0.986) or peak plantar flexor EMG amplitudes during MVIC (26.9 ± 49.5%, t = −1.83, *P* = 0.09) after completing the passive stretching tests. In addition, no correlations were observed between the number of stretching trials (all stretching velocities) and changes in MVIC (*r* = −0.194 [−0.64 to 0.35], *P* = 0.49; Appendix S4; Figure 1A). In Experiment 2, no changes in MVIC (−3.6 ± 7.9%, t = 1.86, *P* = 0.085) or peak plantar flexor EMG amplitudes during MVIC (5.8 ± 34.2%, t = 0.31, *P* = 0.763) were detected after completing the active ROM_max_ stretching tests.

No significant changes were detected in joint moment at 80% of ROM_max_ (F_(2,28)_ = 0.65, *P* = 0.53) or MAC stiffness measured in the last 10° ROM from the joint position reached at 80% of ROM_max_ achieved in the 5° s^−1^ stretching test (F_(2,28)_ = 0.19, *P* = 0.83). No significant changes were detected in peak plantar flexor (F_(2,28)_ = 2.67, *P* = 0.87) or TA (F_(1.15,16.08)_ = 1.23, *P* = 0.29) EMG amplitudes measured in the last 2° ROM from the joint position reached at 80% of ROM_max_ achieved.

#### 3.5.2 Session 2

In Experiment 1, paired‐samples’ t tests revealed no significant changes in MVIC (−1.2 ± 13.3%, t = 0.84, *P* = 0.41) or peak plantar flexor EMG amplitudes during MVIC (17.60 ± 37.15%, t = 1.06, *P* = 0.31) after completing the passive stretching tests. In addition, no correlations were observed between the total number of stretching trials (all stretching velocities) and changes in MVIC (*r* = −0.35 [−0.73 to 0.19], *P* = 0.196; Appendix S4, Figure 1B). In Experiment 2, no changes in maximal voluntary isometric moment (−0.8 ± 14.0%, t = 0.312, *P* = 0.76) or peak plantar flexor EMG amplitudes during MVIC (19.2 ± 80.0%, t = −0.45, *P* = 0.66) were detected after completing the active ROM_max_ stretching tests.

No significant changes were found in joint moment at 80% of ROM_max_ (F_(2,28)_ = 2.05, *P* = 0.15) or MAC stiffness measured in the last 10° ROM from the joint position reached at 80% of ROM_max_ achieved in the 5° s^−1^ stretching test (F_(2,28)_ = 1.02, *P* = 0.37). No significant changes were detected in peak plantar flexor (F_(1.29,18.13)_ = 1.93, *P* = 0.18) or TA (F_(2,28)_ = 2.33, *P* = 0.12) EMG amplitudes measured in the last 2° ROM from the joint position reached at 80% of ROM_max_ achieved.

## 4 DISCUSSION

The present study examined the relative and absolute between‐day (test‐retest) reliabilities of dorsiflexion ROM_max_, peak joint moment during stretches, elastic energy, and musculo‐articular stiffnesses calculated at different ranges of the joint moment‐angle relations obtained from a test battery designed to stretch the plantarflexor muscles to maximum dorsiflexion range of motion at different (stretching) velocities and under different levels of voluntary force using a commercially available dynamometer. We also tested whether the data obtained in either faster or voluntarily active stretches revealed different “flexibility” information than the traditional, slow‐velocity passive stretching test from correlations and cohort‐rank changes between tests, that is, whether there is a methodological (reliability) and information‐driven (similar outcomes and conclusions) benefit of testing an individual under different conditions. The results showed that passive stretch tests could be performed at greater speeds than previously reported with relative reliability ranging from moderate to good based on ICCs. Partially in agreement with our hypothesis, the relative reliability related to ROM_max_ from faster plantar flexor stretch tests was “good” (ICC: 0.79 and 0.87; CVs ranging 3.35‐3.75%), whereas “moderate” relative reliabilities (ICC: 0.54; CV = 9.97%) were observed from the more commonly used slow‐speed test, although approximately similar, moderate to good, reliabilities were observed from plantar flexor mechanical properties between test velocities (see Table 2 for detail). This is an important finding since the effects of tissue viscoelasticity and afferent (reflex) feedback should be greater under faster stretching conditions and are relevant to performances in many activities of daily living, rehabilitation exercises, and sporting tasks.^21^, ^22^ A second important finding that partially supported our second hypothesis was that active muscle stretch tests could be completed with poor to excellent levels of relative reliability in ROM_max_ and mechanical property variables for all variables. In particular, slightly lower ROM_max_ absolute reliabilities (CV: 6.6 and ~8%) in the active than passive tests (see Table 5 for detail), but only when performed at the slowest velocity (5° s^−1^); thus, even with two extensive familiarization sessions, the participants were unable to reliably perform active ROM_max_ plantar flexor stretches at 30 or 60° s^−1^. Since muscles often elongate while active in many activities, the slow‐velocity, active muscle stretch test may provide information relating to tissue properties and ROM_max_ more similar to those observed in daily, occupational, or sporting situations. Of further importance, the findings that passive ROM_max_ tests at faster stretching speeds (≥30° s^−1^) or with muscles voluntarily active provided different (ie, new) information to slow, passive stretches, as evidenced by the between‐test variability in the rank of participants within the cohort (see Appendix S3, Figures 1 and 2; and Table 3 and Figure 3 herein), supported our third hypothesis. This difference is important because conclusions made from test outcomes are usually based on an individual's score relative to a cohort (sample) or population, and a change in an individual's rank within a cohort or population would affect the conclusions drawn from the test. Thus, to gain insight into an individual's maximum ROM capacity or tissue stiffness characteristics under varying conditions, tests under each condition appear to be required. Importantly, the present, extended test battery was completed within a single testing session with no detectible negative effects on neuromuscular performance or MAC mechanical properties, in support of our fourth hypothesis; that is, the completion of each test did not meaningfully influence the outcomes of subsequent tests within the battery.

In the present study, overall ICC_2,1_ values (between‐day relative reliability) for ROM_max_ during passive stretches ranged 0.54–0.87, while SEM values (indicating between‐day absolute reliability) ranged 1.3–4.2°, suggesting moderate to good reliability across variables (Table 2). Additionally, no significant systematic between‐day differences were observed, although ROM_max_ in the 60° s^−1^ test and MAC stiffness from 0°–ROM_max_ at 30° s^−1^ may be considered practically meaningful, that is, 1.5° and 0.5 Nm ^−1^. Moreover, both within‐ and between‐day ROM_max_ reliabilities were greater in the fast‐velocity tests than the more commonly used slow‐velocity test, which was an unexpected but clinically important finding. Because this is the first study to test the reliability of ROM_max_ in plantar flexor stretches performed at faster velocities on an isokinetic dynamometer, it is not possible to compare the present results to previous research findings. Nonetheless, while the present results are consistent with ROM_max_ data obtained in slow‐velocity tests by some researchers,^32^ our reliability values appear slightly lower than the ICC values of ~0.85–0.95 reported by other researchers^33^, ^34^, ^35^ despite our use of multiple familiarization sessions. These differences might be partly explained by methodological differences, such as instructions to participants and/or the criteria adopted for ROM_max_ analysis (see effect of criteria in Appendix S5). Regarding methodological differences, in the present study participants were instructed to cease the stretch at a point where they could “no longer tolerate further stretch” while several other studies required participants to perform stretches to a point of discomfort and/or onset of stretch‐induced pain.^33^, ^34^, ^36^, ^37^, ^38^ Beltrão*, et al* ^32^ showed slightly lower ICC and wider confidence intervals when ROM_max_ was taken at the maximum tolerable sensation of discomfort (ICC_2,3_ = 0.87) than at the angle of first sensation of discomfort (ICC_2,3_ = 0.90). Because our instructions are likely to be more related to maximum tolerable discomfort, we speculate that instructions might have had an effect. Future research should investigate whether instructions given to the participants notably affect ROM_max_ (stretching perception) reliability in tests of different speeds. A second important consideration is that multiple ICC calculation methods exist, and these are not always explicitly detailed by researchers. In the present study, the ICC_2,1_ random‐effect absolute agreement calculation was used; however, higher values would be obtained using other models; for example, for ROM_max_ in the 30 and 60° s^−1^ tests, ICC_2,1_ was 0.79 [95%CI 0.50–0.93] and 0.87 [0.48–0.96] but an ICC_3,1_ mixed‐effect, consistency value would yield 0.82 [0.55–0.94] and 0.92 [0.77–0.97]. However, this might not explain ROM_max_ ICC differences in the slow‐velocity test observed in the present study compared with others since ICC_2,1_ and ICC_3,1_ values were similar. Regardless, some confidence in the test battery might be taken from the findings that future researchers may find higher, rather than lower, reliability values, so our values do not seem to represent an unlikely “best‐case” testing scenario.

A clinically important finding of the present study was that passive stretches performed at higher velocities not only resulted in different ROM_max_, passive moment, elastic energy and MAC stiffness outcomes but also ranked individuals differently within the cohort compared to the slow‐speed stretch (see Tables 1 and 3). In addition, only moderate Spearman's rank‐order correlations were found between dependent variables calculated during stretches performed at 5 and 30° s^−1^ and 5 and 60° s^−1^ (Figures S1 and S2 in Appendix S3). These results were further confirmed by the variability of individual changes in the cohort ranks (relative to the average cohort value) between stretching velocities, that is, Z scores, a method used in previous research.^23^ An individual's change in Z score between tests indicates how many standard deviations (SDs) that individual moved within the cohort relative to the average for two different stretching velocities, although it may not clearly evidence the absolute or relative changes in rank between tests. For example, one participant was ranked 0.37 SD below the average for ROM_max_ at 5° s^−1^ but 1.72 SD below at 30° s^−1^, and then ranked the 10th highest in ROM_max_ at 5° s^−1^ but lowest 30° s^−1^ within the cohort, that is, an absolute change in eight places (53.3% of the cohort) between tests. On the other hand, another participant was ranked 1.04 SD below average at 5° s^−1^ and 1.6 SD below average at 30° s^−1^, but the absolute change in rank was only one place (6.7%; third lowest vs. second lowest). While there was a greater difference in the deviation to the mean between trials, the rank in scores (ie, position within the cohort) did not change in the same proportion. Therefore, the interpretation of both sets of results provides a better general understanding as to how participants changed relative to the mean and how participants changed ranks between tests at different velocities. Overall, these results clearly indicate that the tests performed at faster velocities provide different (ie, new) information than tests performed at the slow velocity; thus, higher‐speed tests are needed in order to determine a person's flexibility status at these speeds.

An important aspect of the present study was the development of a method of testing flexibility while individuals performed a voluntary agonist muscle contraction. We found that the test could be reliably performed at the slow (5° s^−1^) velocity but not at faster velocities (the 30° s^−1^ test was found to be unreliable in Sessions 1 and 2, and the 60° s^−1^ test was abandoned after the familiarization part of the study). Overall, ICC_2,1_ (between‐day reliability) for the slow‐velocity test ranged 0.30‐0.99 across all variables for stretches performed at 40% and 60% of maximal plantar flexion eccentric contraction moment (40‐Ecc and 60‐Ecc, respectively). It was somewhat surprising, but of clinical importance, that an overall “poor to excellent” range for relative reliability and rather smaller CVs and SEMs for most dependent variables was found for data obtained in the active tests but “moderate to good” relative reliability and larger CVs and SEMs were observed in passive, slow velocity, stretching tests. However, despite the “poor to excellent” reliability in active stretching tests, some participants failed to maintain the required joint moment level during the 40‐Ecc because the target joint moment was sometimes too low to be achieved with the muscles active at highly dorsiflexed angles. Although notable ongoing muscle activity was observed at the end of the stretching test, we cannot exclude the possibility that this resulted from reflexive, involuntary muscle activation that is usually observed at greater ranges of motion.^19^ In addition, participants could not clearly subjectively determine whether the test was terminated due to their incapacity to tolerate the stretch (ie, they had reached volitional ROM_max_) or if they were simply unable to maintain the required joint moment level. Out of all participants, only one could explicitly differentiate it, and these data were thus removed from analysis. However, all participants verbally acknowledged that tests performed at 60‐Ecc were terminated because they could not tolerate stretching; this test therefore appears to be a valid ROM_max_ assessment.

ROM_max_ obtained in 40‐Ecc and 60‐Ecc was moderately to strongly correlated with ROM_max_ obtained during slow passive stretches; however, the variability of individual changes in the within‐cohort ranks (Z scores) between tests and the moderate absolute and relative changes in rank between tests indicate that active stretching tests offer different (new) information than the passive, slow‐velocity stretching tests. Thus, active stretching tests are needed in order to determine a person's flexibility status during active stretching tests. Future studies are needed to determine the reliability of tests performed under higher force levels than those used in the present study, and whether temporal differences exist in the responses to active versus passive stretch tests after physical training interventions or alterations in impairment or disease status.

An important final aim of the study was to determine whether the stretching test battery could be completed without the tests themselves influencing neuromuscular performance or MAC mechanical properties. The results did not reveal a stretch‐induced force loss or any changes in passive mechanical properties, as measured by MVC and stretch tests completed at various time points across the experimental sessions. These results are important because several researchers have reported detrimental effects on neuromuscular performance and changes in MAC stiffness after a series of maximal muscle stretches.^25^, ^39^, ^40^ However, these changes occurred after the completion of a higher stretching volume than performed in the present study.^39^, ^40^ This information is suggestive that the current test battery could be completed in its entirety in clinical or sporting testing environments without concern that later tests might be impacted by earlier tests, and further indicates test validity.

## 5 CONCLUSION

The results of the present study demonstrate that the test battery provided poor to excellent test‐retest relative and absolute reliabilities for ankle ROM_max_, peak passive joint moment, MAC stiffness, and passive elastic energy for passive stretching tests performed at all velocities as well as those imposed on active muscles at 40 and 60% of maximal eccentric contraction moment at the slow stretch velocity (5° s^−1^). Of note was that both the faster‐velocity passive tests and the slow‐velocity active test provided slightly overall “better” ranges of reliability than the commonly performed slow‐velocity ankle plantar flexor stretch test. It was also confirmed that “new” and different information (evidenced by correlations as well as changes in individual cohort rank and non‐homogeneous linearity in individual Z scores between tests) was provided by the faster‐velocity passive tests and the active slow‐velocity test than the commonly performed slow‐velocity stretch test. Therefore, a description of an individual's “flexibility” characteristics under conditions where relatively fast stretches are performed or where voluntary muscle contractions are present cannot be accurately determined from tests at slow speeds. Importantly, the present data indicate that the test battery can be completed within a single testing session with no negative consequences for neuromuscular performance or MAC mechanical properties. Thus, using the methods described in the present study, testing of ankle joint ROM, resistance to stretch (ie, stiffness) and other variables that describe and individual's flexibility characteristics may be completed under conditions of different stretch velocity and levels of muscle force production.

## 6 PERSPECTIVES

Maximal joint range of motion and resistance to tissue elongation are important determinants of an individual's ability to perform movements of daily living or sporting tasks, and may be associated with muscle strain injury risk.^1^, ^2^, ^3^, ^4^ These physical characteristics are often assessed in clinical and sports settings by rotating a joint slowly with the muscles voluntarily relaxed.^15^ However, such slow voluntarily relaxed tests may reduce the functional relevance to activities of daily living or sporting tasks, which are performed at faster velocities and usually under the influence of reflexive and/or voluntary muscle activity.^21^ Therefore, new tests need to be developed and tested for reliability to provide greater scrutiny of the relationship between “flexibility” and function in complex human movement. Here, we show that tests not only can be completed with poor to excellent reliability across velocities and force levels, but also offer (new) different information. That is, a description of an individual's “flexibility” characteristics under conditions where relatively fast stretches are performed or where voluntary muscle contractions are present cannot be accurately estimated from the traditional, voluntary‐relaxed, slow‐velocity tests. Future experiments are warranted to determine whether the tests can be completed with high reliability in other populations, including elderly and clinical populations, and whether temporal changes, including physical training and aging, can be tracked with accuracy.

## CONFLICT OF INTEREST

No conflicts of interest, financial, or otherwise are declared by the author(s).

## Supporting information

Appendix S1

Appendix S2

Appendix S3

Appendix S4

Appendix S5

Appendix S6

Appendix S7

Appendix S8

Appendix S9
