# Supplementary figures and images for: Reliability of isokinetic tests of velocity‐ and contraction intensity‐dependent plantar flexor mechanical properties
Source: Scand J Med Sci Sports. 2021 Mar 23;31(5):1009–25. doi: 10.1111/sms.13920 (PMC8251531; doi:10.1111/sms.13920)

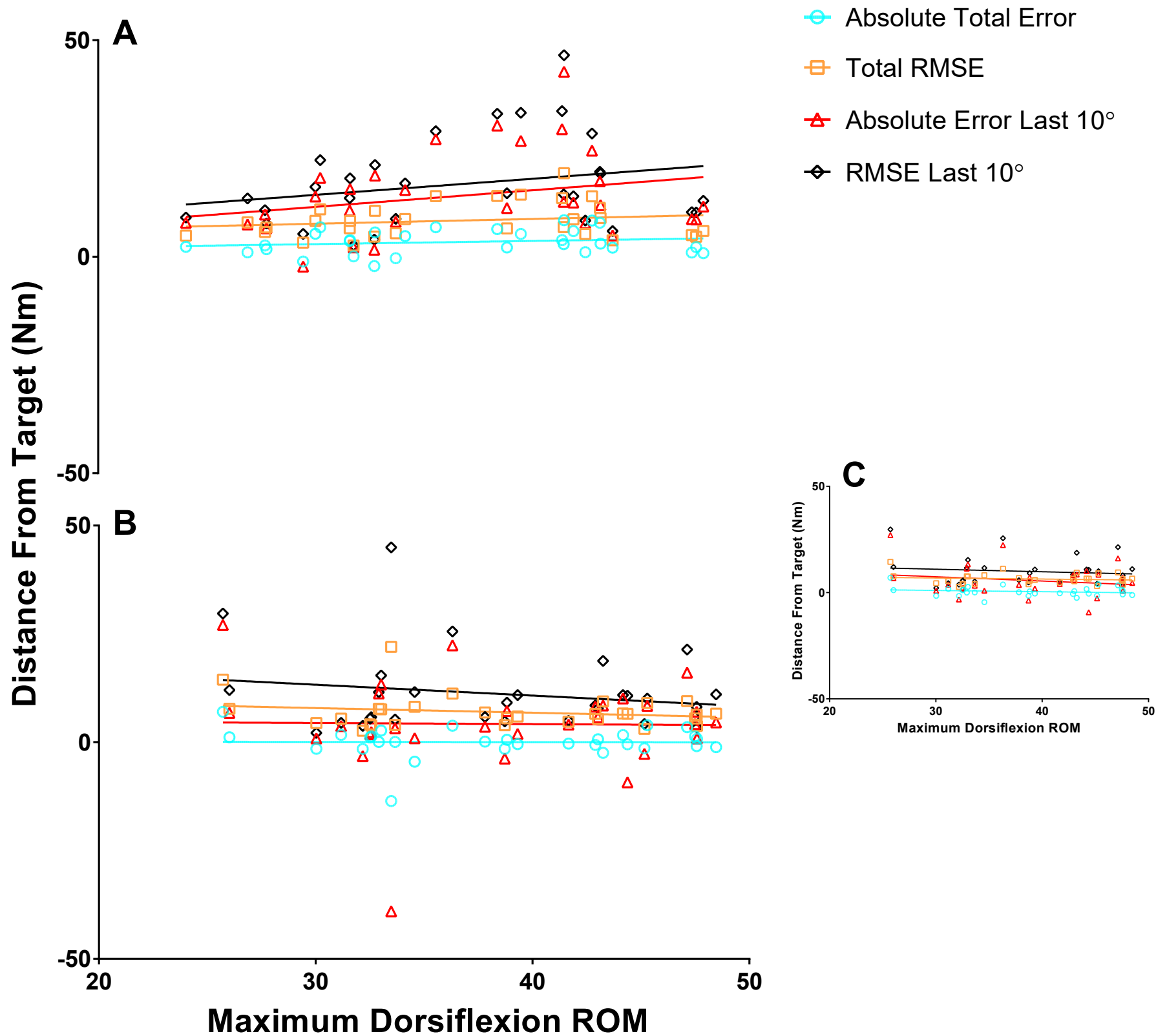

Supplement: Supplementary file 1 — Appendix S1 [file SMS-31-1009-s002.pdf]

A

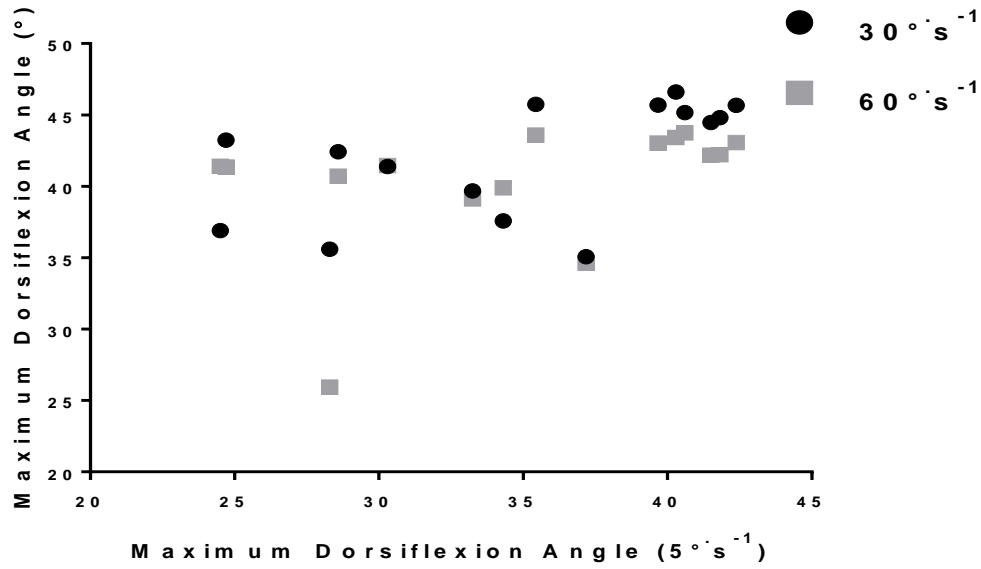

B

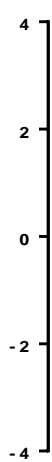

C

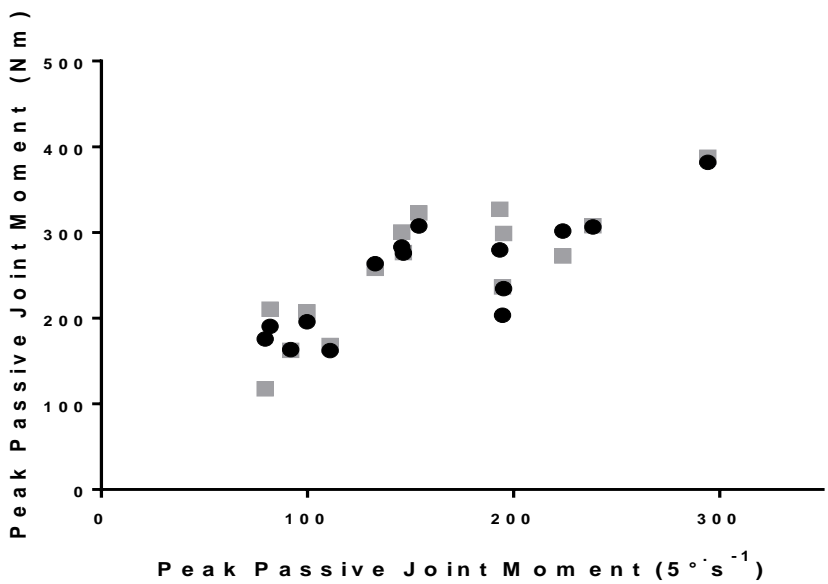

D

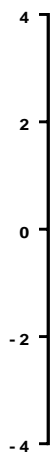

E

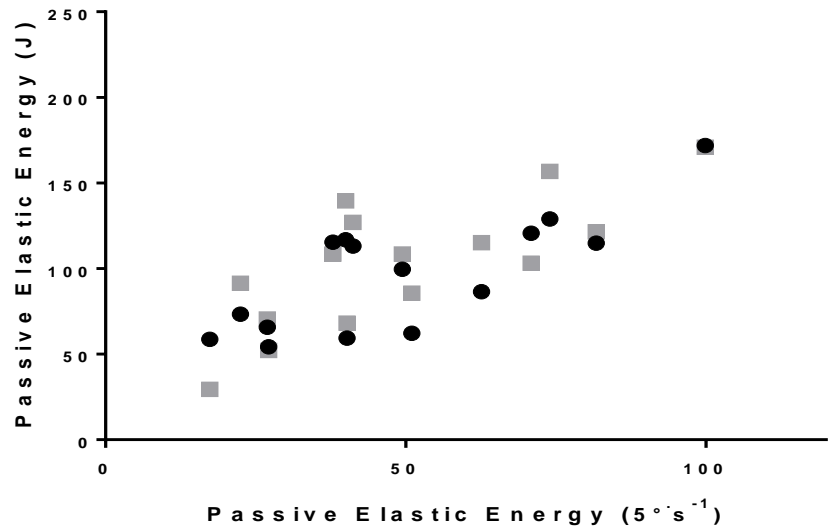

F

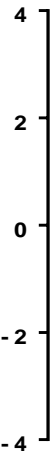

G

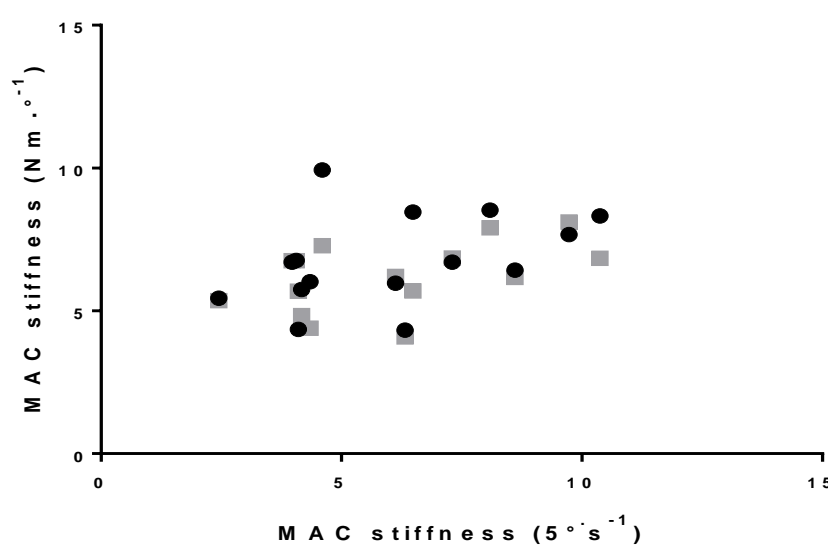

H

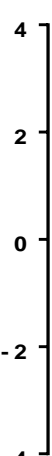

I

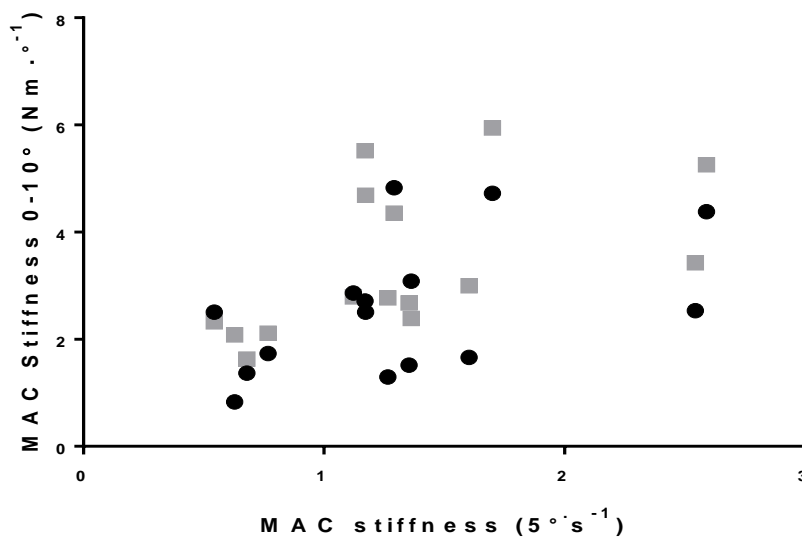

J

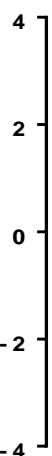

K

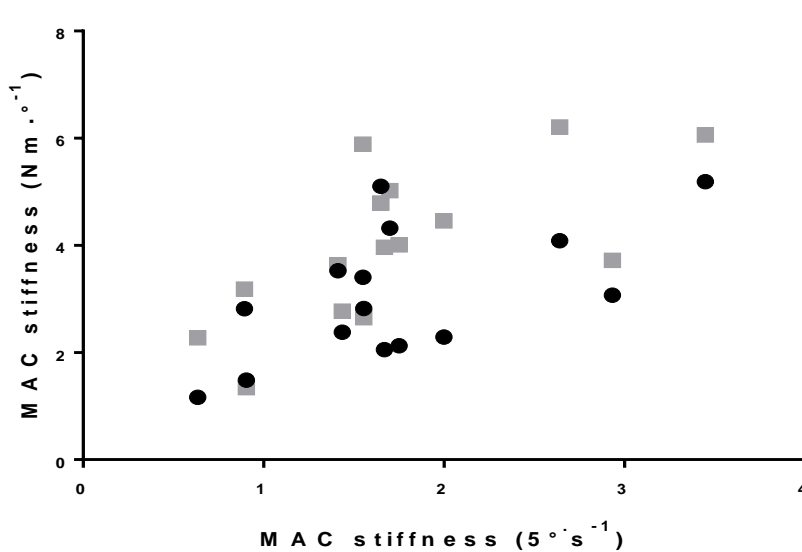

L

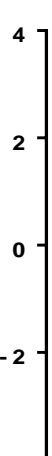

M

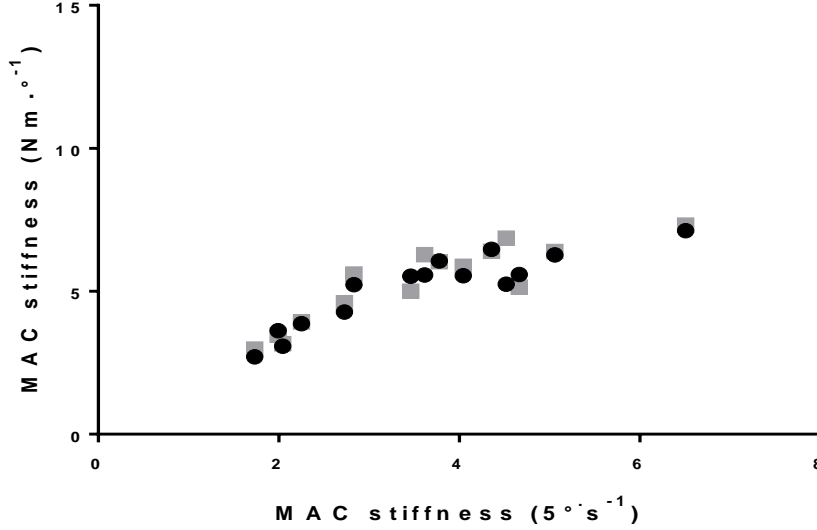

N

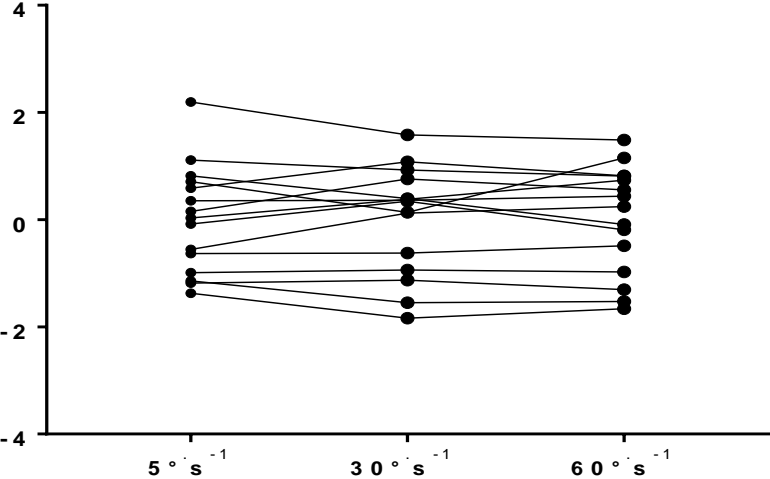

Supplement: Supplementary file 2 — Appendix S2 [file SMS-31-1009-s001.pdf]

**A**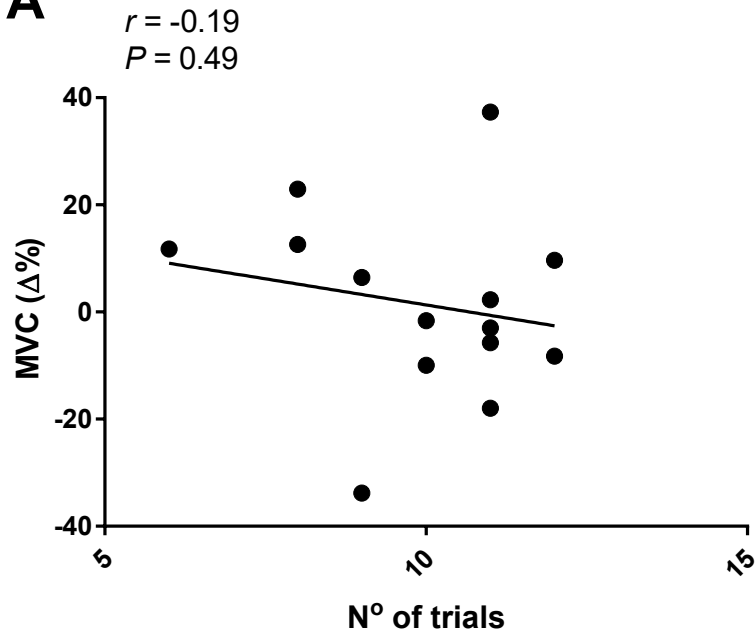**B**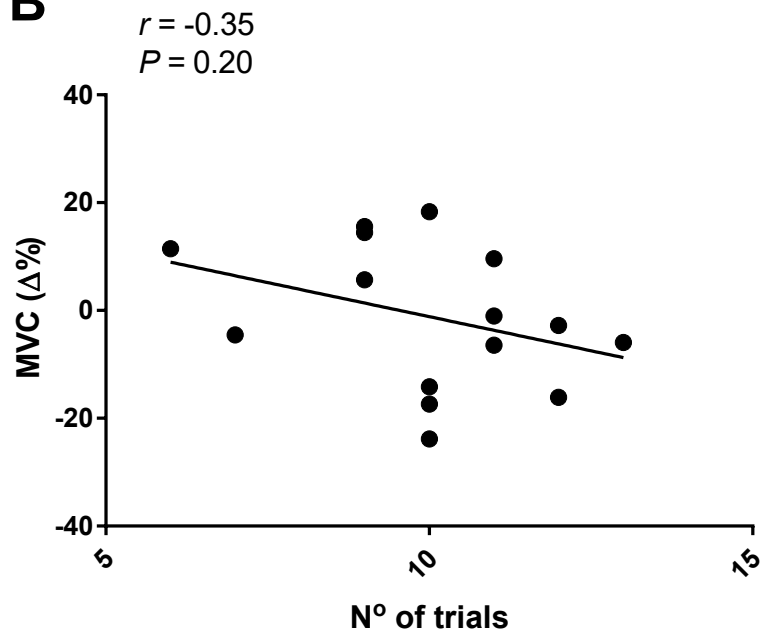

Supplement: Supplementary file 3 — Appendix S3 [file SMS-31-1009-s007.pdf]

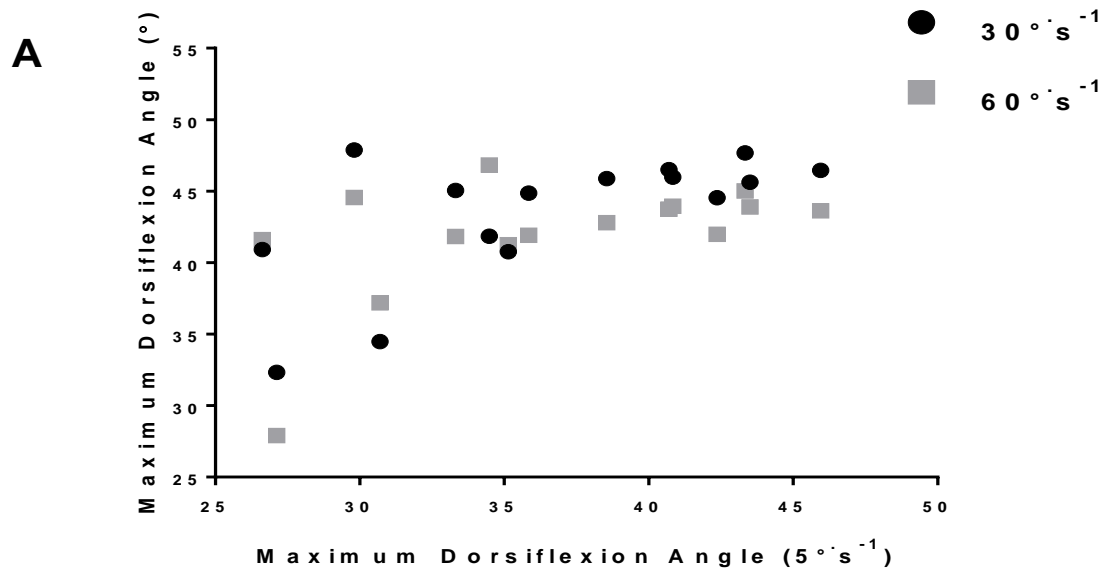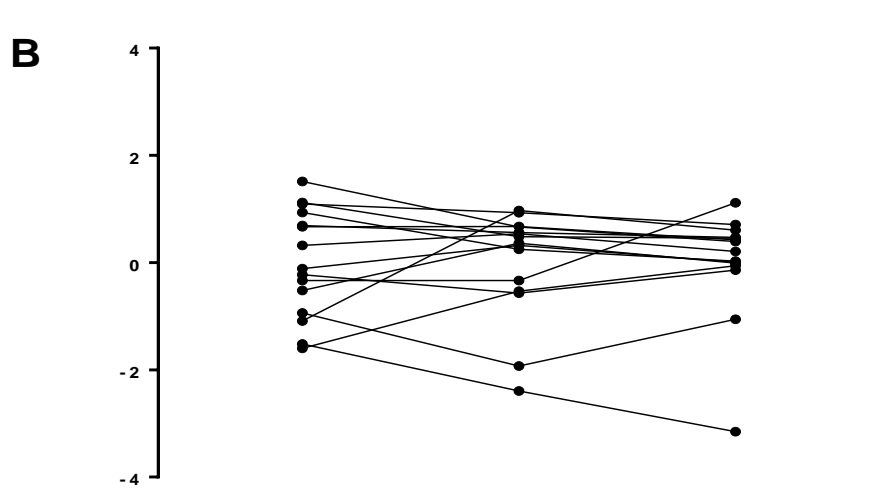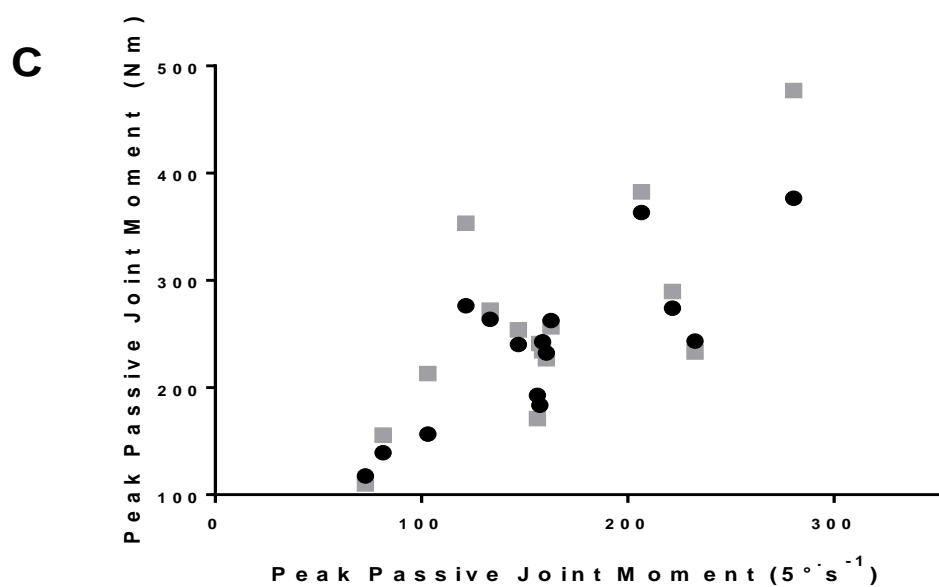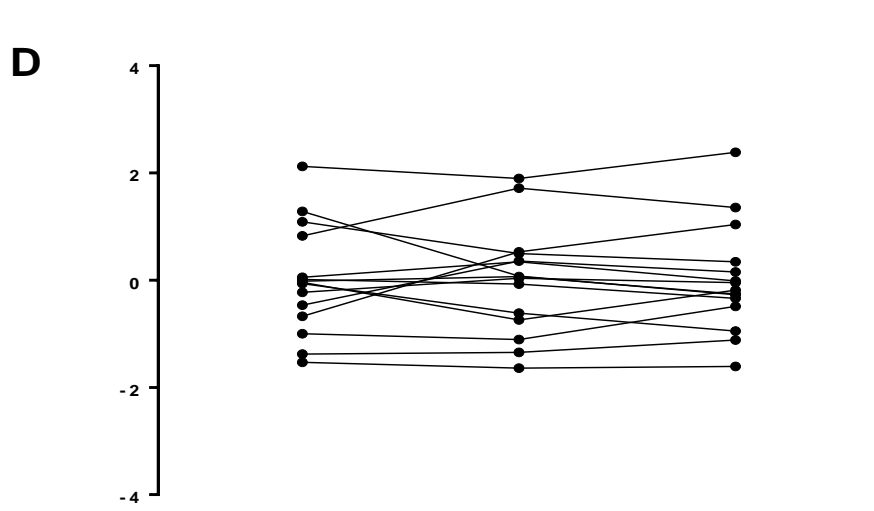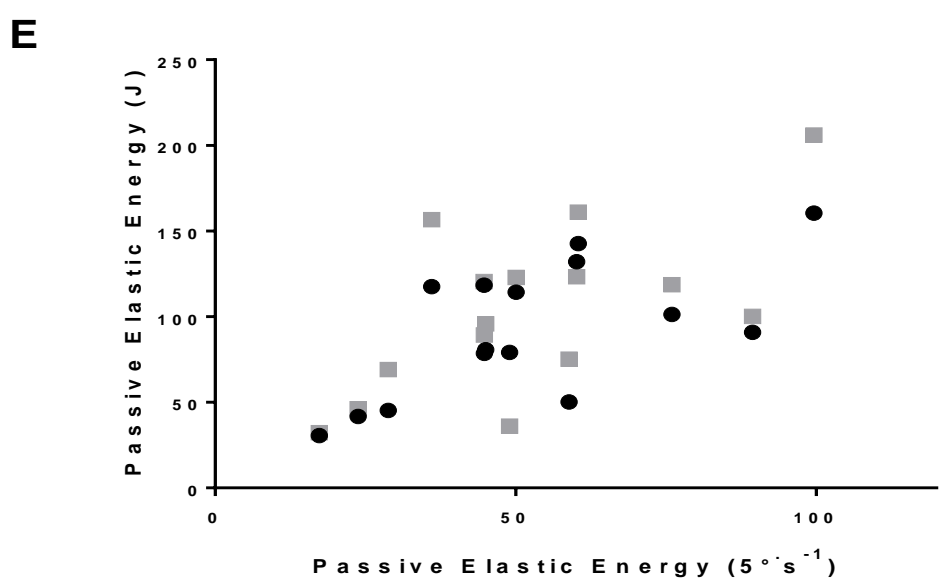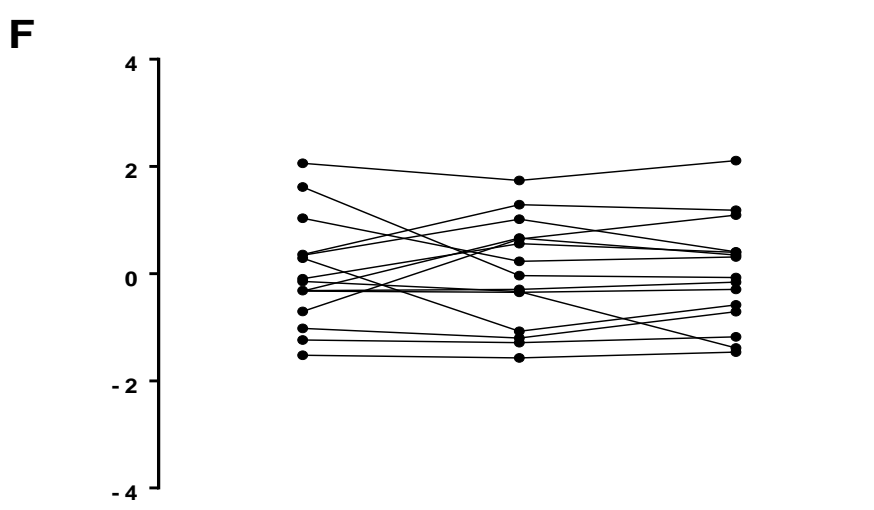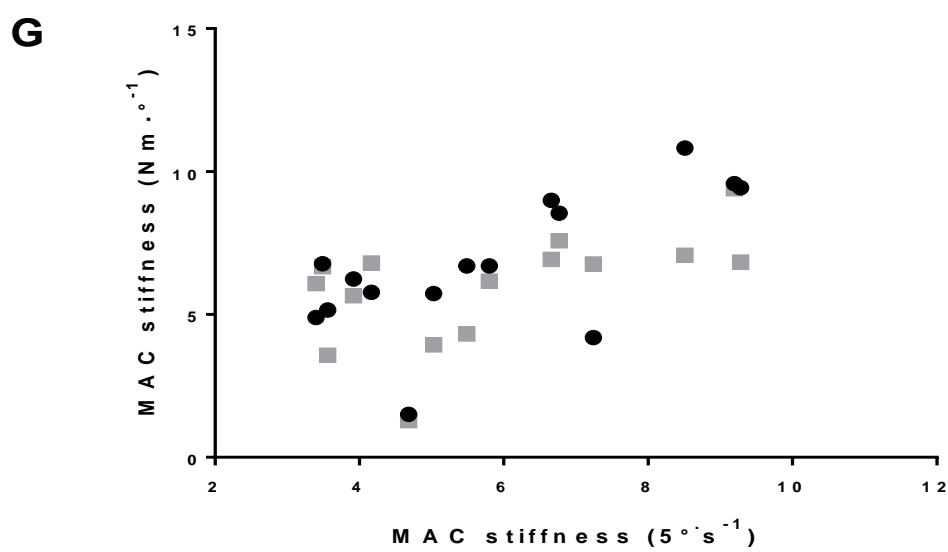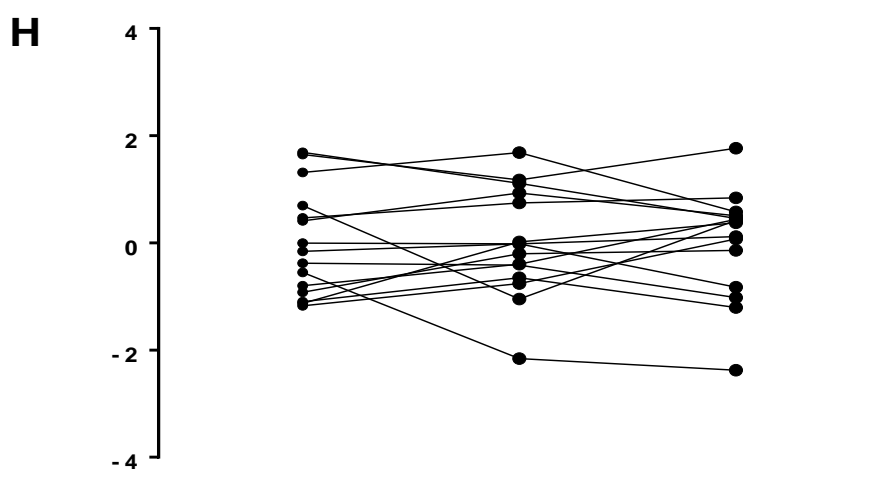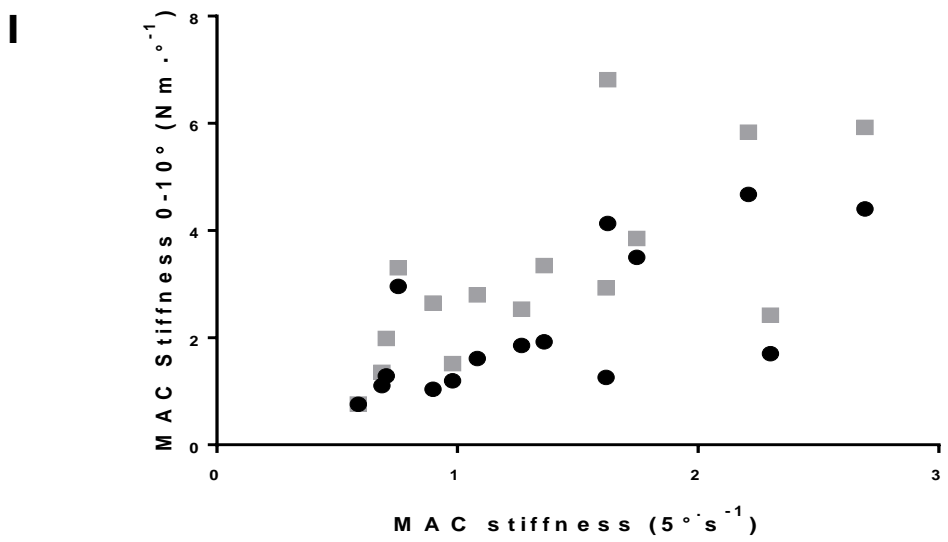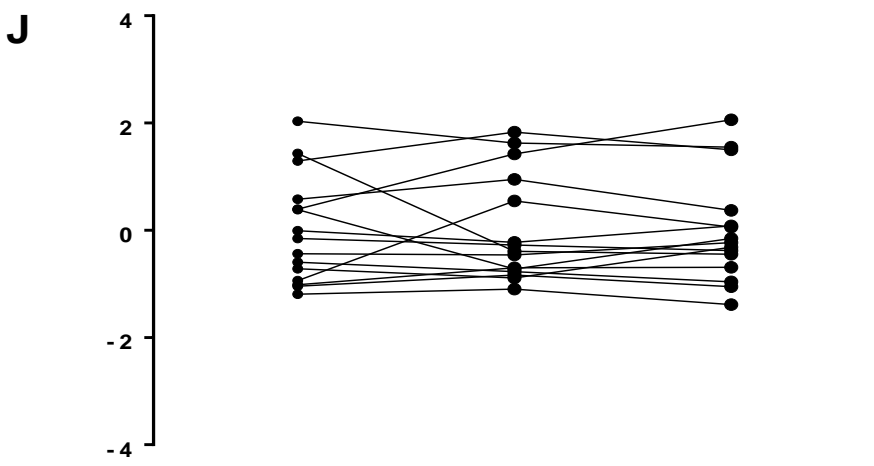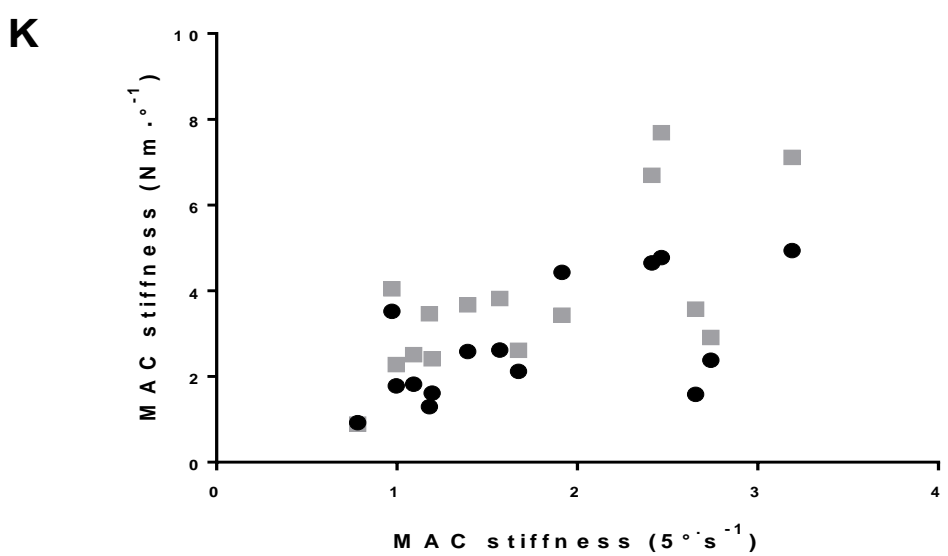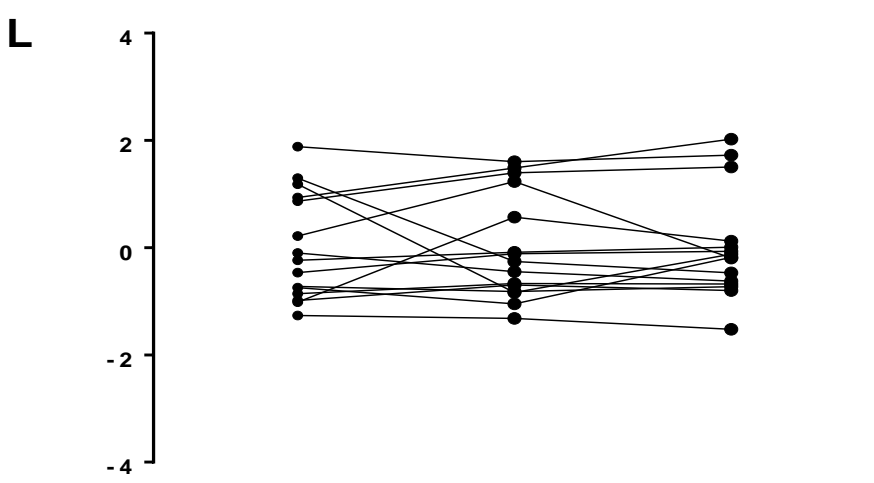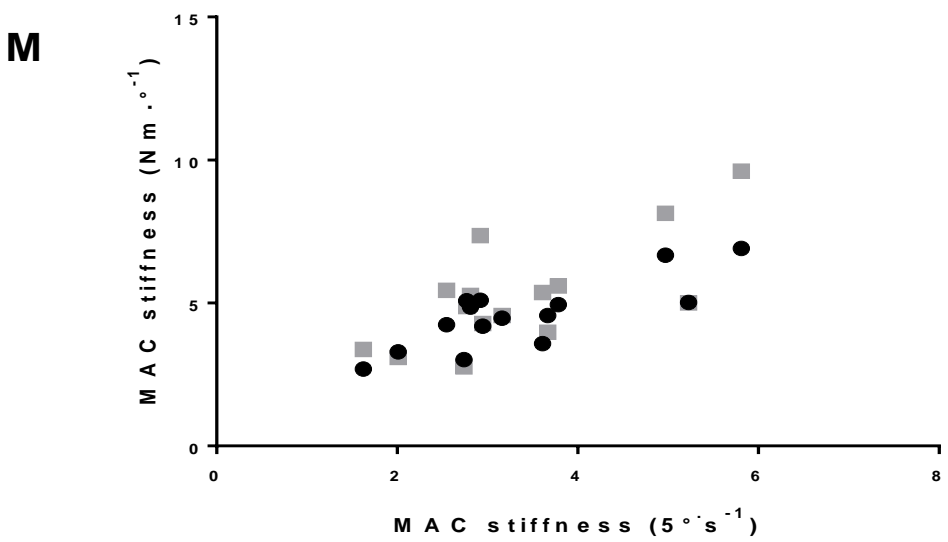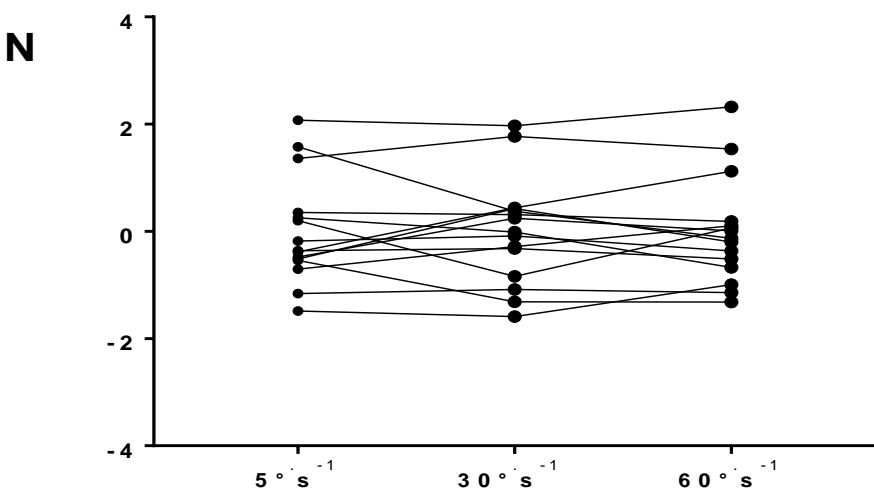

Supplement: Supplementary file 4 — Appendix S4 [file SMS-31-1009-s009.pdf]
