# Supplementary material for: Reliability of isokinetic tests of velocity‐ and contraction intensity‐dependent plantar flexor mechanical properties
Source: Scand J Med Sci Sports. 2021 Mar 23;31(5):1009–25. doi: 10.1111/sms.13920 (PMC8251531; doi:10.1111/sms.13920)
Supplement: Supplementary file 5 — Appendix S5 [file SMS-31-1009-s008.docx]

**SUPPLEMENTAL MATERIAL 1**

| **Table 1.** Relative and absolute (test-retest) reliability analyses completed with the participant removed for passive stretches. Intra-class correlation coefficient (ICC_2,1_) indicates relative reliability whilst standard error of measurements (SEM, i.e. typical error), coefficients of variation (CV%), and minimal detectable changes (MDC) indicate absolute reliability. | | | | | | | | | | | | | | |
| --- | --- | --- | --- | --- | --- | --- | --- | --- | --- | --- | --- | --- | --- | --- |
|  | Passive ankle joint rotation velocities | | | | | | | | | | | | | |
|  | 5°·s^-1^ | | | |  | 30°·s^-1^ | | | |  | 60°·s^-1^ | | | |
|  | ICC (95% CI) | SEM | CV (%) | MDC |  | ICC (95% CI) | SEM | CV (%) | MDC |  | ICC (95% CI) | SEM | CV (%) | MDC |
| Maximum dorsiflexion angle (°) | 0.57 (0.11 to 0.83) | 3.93 | 9.41 | 7.7 |  | 0.80 (0.48 to 0.93) | 1.86 | 3.60 | 3.64 |  | 0.88 (0.52 to 0.97) | 1.35 | 3.38 | 2.64 |
| Peak passive joint moment (Nm) | 0.69 (0.25 to 0.89) | 35.5 | 17.3 | 69.6 |  | 0.75 (0.39 to 0.91) | 36.0 | 13.0 | 70.5 |  | 0.83 (0.54 to 0.94) | 35.6 | 9.65 | 69.8 |
| Passive elastic energy (J) | 0.70 (0.30 to 0.89) | 13.5 | 21.7 | 26.4 |  | 0.85 (0.59 to 0.95) | 14.9 | 15.8 | 29.2 |  | 0.87 (0.63 to 0.96) | 16.6 | 14.4 | 32.5 |
| MAC stiffness (Nm·°^-1^) |  |  |  |  |  |  |  |  |  |  |  |  |  |  |
| 0 – 10° | 0.84 (0.56 to 0.94) | 0.26 | 17.2 | 0.52 |  | 0.81 (0.48 to 0.93) | 0.48 | 24.51 | 0.93 |  | 0.75 (0.39 to 0.91) | 0.77 | 22.7 | 1.51 |
| 0 – 20° | 0.83 (0.54 to 0.94) | 0.33 | 18.3 | 0.65 |  | 0.73 (0.37 to 0.90) | 0.62 | 23.2 | 1.22 |  | 0.81 (0.52 to 0.93) | 0.71 | 17.3 | 1.38 |
| Last 10° | 0.59 (0.09 to 0.85) | 1.48 | 21.3 | 2.90 |  | 0.64 (0.17 to 0.87) | 1.30 | 16.0 | 2.56 |  | 0.71 (0.33 to 0.90) | 0.92 | 14.7 | 1.80 |
| 0° – ROM_max_ | 0.72 (0.33 to 0.90) | 0.70 | 16.9 | 1.38 |  | 0.72 (0.32 to 0.90) | 0.63 | 10.9 | 1.23 |  | 0.75 (0.40 to 0.91) | 0.83 | 10.6 | 1.63 |

| **Table 2.** Relative and absolute (test-retest) reliability analyses completed with the participant removed for active stretching tests performed at 40- and 60-Ecc. Intra-class correlation coefficient (ICC_2,1_) indicates relative reliability, whilst standard error of measurements (SEM, i.e. typical error), coefficients of variation (CV%), and minimal detectable changes (MDC) indicate absolute reliability. | | | | | | | | | | |
| --- | --- | --- | --- | --- | --- | --- | --- | --- | --- | --- |
|  | Ankle joint moment feedback for stretching tests performed at 5°.s^-1^ | | | | | | | | | |
|  | 40-Ecc | | | |  | 60-Ecc | | | | |
|  | ICC (95% CI) | SEM | CV (%) | MDC |  | ICC (95% CI) | SEM | CV (%) | MDC |  |
| Maximum dorsiflexion angle (°) | 0.67 (0.21 to 0.89) | 3.61 | 7.25 | 7.10 |  | 0.70 (0.30 to 0.89) | 3.59 | 6.76 | 7.04 |  |
| Peak passive joint moment (Nm) | 0.76 (0.38 to 0.92) | 20.9 | 7.9 | 41.0 |  | 0.92 (0.77 to 0.97) | 13.7 | 3.92 | 26.8 |  |
| Passive elastic energy (J) | 0.79 (0.43 to 0.93) | 13.0 | 11.3 | 25.5 |  | 0.86 (0.61 to 0.95) | 16.7 | 8.76 | 32.7 |  |
| Musculo-articular complex stiffness (Nm·°^-1^) |  |  |  |  |  |  |  |  |  |  |
| 0 – 10° | 0.30 (-0.27 to 0.66) | 0.53 | 20.7 | 1.03 |  | 0.55 (0.28 to 0.83) | 0.66 | 16.3 | 1.29 |  |
| 0 – 20° | 0.93 (0.80 to 0.98) | 0.14 | 6.49 | 0.27 |  | 0.86 (0.61 to 0.95) | 0.29 | 6.4 | 0.58 |  |
| Last 10° | 0.67 (0.20 to 0.89) | 1.50 | 23.3 | 2.94 |  | 0.44 (-0.13 to 0.78) | 1.75 | 173.6 | 3.42 |  |
| 0° – ROM_max_ | 0.72 (0.29 to 0.91) | 0.53 | 16.4 | 1.03 |  | 0.99 (0.95 to 0.97) | 0.11 | 2.98 | 0.21 |  |
