# Supplementary material for: Reliability of isokinetic tests of velocity‐ and contraction intensity‐dependent plantar flexor mechanical properties
Source: Scand J Med Sci Sports. 2021 Mar 23;31(5):1009–25. doi: 10.1111/sms.13920 (PMC8251531; doi:10.1111/sms.13920)
Supplement: Supplementary file 6 — Appendix S6 [file SMS-31-1009-s006.docx]

**SUPPLEMENTAL MATERIAL 2**

**Correlation between active joint moment variability and error during active muscle stretches and the maximum range of motion achieved**

**Background:** In Experiment 2, the participants performed active maximum range of motion (ROM_max_) stretching tests at 5°^.^s^-1^ whilst maintaining contraction intensities of 40% (±5 Nm; Ecc-40) and then 60% (±5 Nm; Ecc-60) of maximum voluntary eccentric contraction moment (Ecc-MVC) of the best trial achieved in the familiarisation session 1 or 2. During the active stretching tests, participants were instructed to maintain the joint moment level between the guidelines set on a computer placed in front of them throughout the ROM (see Fig. 2 and main text for details). Participants volitionally terminated the stretching test by pressing a handheld button at a “point where they could no longer tolerate being stretched” or when they could not self-adjust their voluntary moment within the guidelines, i.e. target moment. Despite participants being extensively familiarised with the testing protocol, joint moments sometimes fluctuated outside of the target during the stretching test. It was therefore necessary to determine whether small fluctuations substantially impacted results, or could otherwise be tolerated in the test.

To assess this, mean absolute and root mean square errors (RMSE) from the target were calculated and correlated with ROM_max_ from 40-Ecc and 60-Ecc stretching tests to determine whether these fluctuations were associated with a greater or lesser ROM_max_. Trials with greatest and least ROM_max_ were selected randomly from Sessions 1 or 2, and errors were calculated throughout the stretching trial as well as only in the last 10° of ROM (the range with greatest deviations from target and most likely to influence ROM_max_).

Kolmogorov-Smirnov testing showed that all dependent variables at 40-Ecc were normally distributed, whilst four variables were not normally distributed for 60-Ecc. Significant outliers (3.27 standard deviations above or below the average) were excluded before Pearson’s product-moment correlation analyses were performed between ROM_max_ and joint moment error. As shown in Figure 1A, in the 40-Ecc stretching tests there were no significant correlations between ROM_max_ and absolute and root mean square errors calculated throughout the stretching trial (r = 0.18 (-0.20 to 0.50), *P* = 0.35; and r = 0.19 (-0.19 to 0.51), *P* = 0.32) or in the last 10° of ROM (r = 0.27 (-0.10 to 0.57), *P* = 0.15; and r = 0.24 (-0.13 to 0.56), *P* = 0.19). Additionally, Pearson’s product-moment correlation analysis revealed no significant correlations between ROM_max_ and absolute and root mean square errors calculated throughout the 60-Ecc stretching trial (r = -0.008 (-0.37 to 0.35), *P* = 0.96; and r = -0.19 (-0.52 to 0.18), *P* = 0.31) or in the last 10° of ROM (r = -0.016 (-0.37 to 0.35), *P* = 0.93; and r = -0.19 (-0.51 to 0.19), *P* = 0.33). These results are graphically represented in Figure1B. We also conducted these analyses by removing one significant outlier (n=29), as shown in Figure1C. Pearson’s product-moment relationship again showed no significant correlations between ROM_MAX_ and absolute and root mean square errors calculated throughout the stretching trial (r = -0.18 (-0.51 to 0.20), *P* = 0.35; and r = -0.13 (-0.47 to 0.25), P = 0.49) or in the last 10° of ROM (r = -0.18 (-0.51 to 0.20), P = 0.34; and r = -0.12 (-0.47 to 0.25), *P* = 0.52). Overall, these results indicate that a participant’s ability to maintain the joint moment within the target was not associated with the ROM_max_ achieved during the test ROM_max_.

**Figure 1.** Relationships between mean absolute and root mean square errors (RMSE) from the target and maximal volitional dorsiflexion range of motion (ROM) from active stretching trials performed at 40% (A) and 60% (B, N=30; and C, outlier removed, n=29) of maximal eccentric voluntary contractions. No significant relationships were found.
