# Supplementary material for: Reliability of isokinetic tests of velocity‐ and contraction intensity‐dependent plantar flexor mechanical properties
Source: Scand J Med Sci Sports. 2021 Mar 23;31(5):1009–25. doi: 10.1111/sms.13920 (PMC8251531; doi:10.1111/sms.13920)
Supplement: Supplementary file 7 — Appendix S7 [file SMS-31-1009-s004.docx]

**SUPPLEMENTAL MATERIAL 2**

**Associations between variables calculated from data collected in passive stretch tests (Experiment 1) at different velocities**

**Background:** A laboratory- or clinic-based set of tests that allows for the assessment of ROM_max_ and musculoarticular (MAC) mechanical properties at faster joint rotation velocities and higher levels of voluntary force was developed in order to provide greater scrutiny of the relationship between ‘flexibility’ and function in complex human movement tasks, as well as to track the potential changes in flexibility under conditions more similar to those faced during daily or sporting activities. For this test battery to be practically and clinically meaningful, the information given by the higher-velocity or active muscle tests should differ from that provided by the traditional low-velocity, passive (i.e. standard) tests. That is, in addition to giving different scores of ‘flexibility’, it would also have to rank individuals differently within a cohort. This is important because conclusions made from test outcomes are usually based on an individual’s score relative to a cohort (sample) or population, and a change in an individual’s rank within a cohort or population would affect the conclusions drawn from the test.

Thus, we determined whether the ‘flexibility’ variables commonly calculated in previous studies and that are commonly tested in the clinical field ranked individuals differently within the cohort across stretching tests performed at different velocities and force levels. Variables included were passive and active ROM_max_, peak passive and active joint moments (stretch tolerance), areas under the passive and active joint moment-angle relations (elastic potential energy storage), and passive and active joint moment-angle relation gradients (MAC stiffness) calculated in several ranges of the joint-moment angle relations to cover a broad spectrum of joint ROMs achieved in sporting tasks and activities of daily living.

*Session 1*

Spearman’s correlation analysis revealed significant positive moderate-to-strong correlations between ROM_max_ obtained in tests at 5°·s^-1^ and those at 30°·s^-1^ (r*_s_* = 0.59 [95% CI: 0.1 to 0.8], *P* = 0.02) and 60°·s^-1^ (r*_s_* = 0.6 [0.1 to 0.8], *P* = 0.02). However, visual inspection of individual changes revealed no standard pattern of change when rank scores were graphically represented (see Figure 1B). Thus, participants that scored best or worst in ROM_max_ at 5°·s^-1^ did not necessarily maintain this position in joint rotations performed at 30 and 60°·s^-1^. On average, participants were ranked 3.3 (5 vs. 30°·s^-1^), 3.5 (5 vs. 60°·s^-1^) and 1.5 (30 vs. 60°·s^-1^) places different within the cohort, equating to 22.2, 23.1, 9.8% changes in ranking. The maximum changes in rank between tests were 8 (53.3%), 7 (46.7%) and 4 (26.7%), respectively.

Significant strong, positive correlations between peak passive joint moments obtained in tests at 5°·s^-1^ and those at 30°·s^-1^ (r*_s_* = 0.78 [0.4 to 0.9], *P* = 0.001), and 60°·s^-1^ (r*_s_* = 0.78 (95% CI, 0.4 to 0.9, *P* < 0.001). However, visual inspection of individual changes revealed no standard pattern of change when rank scores were graphically represented (see Figure 1D). Thus, a participant’s peak joint moment rank at 5°·s^-1^ was not always similar to their rank in tests at 30 and 60°·s^-1^. On average, participants were ranked 2.3 (5 vs. 30°·s^-1^), 2.3 (5 vs. 60°·s^-1^) and 1.3 (30 vs. 60°·s^-1^) places different within the cohort, equating to 15.1, 15.1, 8.9% changes in ranking. The maximum changes in rank between tests were 5 (33.3%), 5 (33.3%) and 4 (26.6%), respectively.

Significant moderate-to-strong, positive correlations between passive elastic energy obtained in tests at 5°·s^-1^ and those at 30°·s^-1^ (r*_s_* = 0.66 (95% CI, 0.2 to 0.9), *P* = 0.009), and 60°·s^-1^ (r*_s_* = 0.7 (95% CI, 0.3 to 0.9, *P* = 0.005). However, visual inspection of individual changes revealed no standard pattern of change when rank scores were graphically represented (see Figure 1E). Thus, a participant’s passive elastic energy rank at 5°·s^-1^ was not necessarily the same as the rank at 30 and 60°·s^-1^. On average, participants were ranked 2.9 (5 vs. 30°·s^-1^), 2.7 (5 vs. 60°·s^-1^), and 1.5 (30 vs. 60°·s^-1^) places different within the cohort, equating to 19.6, 17.8, 9.8% changes in ranking. The maximum changes in rank between tests were 6 (40%), 7 (46.7%) and 6 (40%), respectively.

No significant correlations were observed between MAC stiffness calculated through the last 10° in tests obtained at 5°·s^-1^ and those at 30°·s^-1^ (r*_s_* = 0.4 (95% CI, -0.1 to 0.8), *P* = 0.1), and 60°·s^-1^ (r*_s_* = 0.4 (95% CI, -0.1 to 0.8, *P* = 0.09). This was further confirmed by visual inspection of individual changes, where no standard pattern of change was observed in rank scores graphically represented (see Figure 1H). On average, participants were ranked 3.9 (5 vs. 30°·s^-1^), 3.7 (5 vs. 60°·s^-1^) and 2.1 (30 vs. 60°·s^-1^) places different within the cohort, equating to 25.8, 24.9, 2.1% changes in ranking. The maximum changes in rank between tests were 8 (53.3%), 8 (53.3%) and 7 (46.7%), respectively.

No significant correlations were observed between MAC stiffness calculated from neutral to 10° (MAC_0-10_) of dorsiflexion in tests at 5 and 30°·s^-1^ (r*_s_* = 0.50 (95% CI, -0.04 to 0.81, P = 0.06). However, a moderate-to-strong correlation was observed between MAC_0-10_ in tests obtained at 5 and 60°·s^-1^ (r*_s_* = 0.64 (95% CI, 0.2 to 0.9, *P* = 0.01). Visual inspection of individual changes showed no standard pattern of change was observed in rank scores graphically represented (see Figure 1J). On average, participants were ranked 3.6 (5 vs. 30°·s^-1^), 3.1 (5 vs. 60°·s^-1^) and 2.9 (30 vs. 60°·s^-1^) places different within the cohort, equating to 24.0, 20.4, 19.6% changes in ranking. The maximum changes in rank between tests were 7 (46.7%), 8 (53.3%) and 7 (46.7%), respectively.

No significant correlations were observed between MAC stiffness calculated from neutral to 20° of dorsiflexion (MAC_0-20_) in tests at 5 and 30°·s^-1^ (r*_s_* = 0.47 (95% CI, -0.07 to 0.80), *P* = 0.08, respectively). However, a significant strong, positive correlation was observed between MAC_0-20_ in tests obtained at 5 and 60°·s^-1^ (r*_s_* = 0.71 (95% CI, 0.3 to 0.7, *P* = 0.004). Visual inspection of individual changes showed no standard pattern of change was observed in rank scores graphically represented (see Figure 1L). On average, participants were ranked 3.6 (5 vs. 30°·s^-1^), 2.7 (5 vs. 60°·s^-1^) and 2.9 (30 vs. 60°·s^-1^) places different within the cohort, equating to 24.0, 17.8, 19.6% changes in ranking. The maximum changes in rank between tests were 7 (46.7%), 7 (46.7%) and 5 (33.3%), respectively.

Significant strong, positive correlations between MAC stiffness calculated from 0° to ROM_max_ (MAC_0–ROMmax_) obtained in tests at 5 and 30°·s^-1^ (r*_s_* = 0.90 (95% CI, 0.7 to 0.9), *P <* 0.001), and 60°·s^-1^ (r*_s_* = 0.88 (95% CI, 0.7 to 1.0, *P* < 0.001). However, visual inspection of individual changes revealed no standard pattern of change when rank scores were graphically represented (see Figure 1N). On average, participants were ranked 1.3 (5 vs. 30°·s^-1^), 1.5 (5 vs. 60°·s^-1^) and 1.3 (30 vs. 60°·s^-1^) places different within the cohort, equating to 8.9, 9.8, 8.9% changes in ranking. The maximum changes in rank between tests were 5 (33.3%), 6 (40.0%) and 7 (46.7%), respectively.

**Figure 1.** ***Left panel (A, C, E, G, I, K, M):*** Relationships between maximum joint range of motion, peak passive joint moment, passive elastic energy, MAC stiffness calculated through the last 10°, from neutral to 10° and 20° of dorsiflexion, and from 0° to ROM_max_ at 5°·s^-1^ (x-axis), and those at 30 and 60°·s^-1^ (y-axis) from Session 1. Overall, moderate-to-strong significant relationships were found between variables from tests performed at 5°·s^-1^ and those at 30 or 60°·s^-1^. ***Right panel (B, D, F, H, J, L, N):*** Individual relative (to the cohort mean, Z scores) change in scores for each dependent variable obtained at each joint rotation velocities. The non-standard changes in scores obtained at each joint rotation velocity suggest that different conclusions as to an individual’s capacity might be drawn from these tests.

*Session 2*

Spearman’s correlation analysis revealed significant moderate-to-strong, positive correlations in ROM_max_ obtained in tests at 5 and 30°·s^-1^ (r*_s_* = 0.52 [95% CI: 0.0007 to 0.82], *P* = 0.047), but no significant correlations were observed in ROM_max_ obtained in tests at 5 and 60°·s^-1^ (r*_s_* = 0.50 [-0.05 to 0.80], *P* = 0.07). Visual inspection of individual changes revealed no standard pattern of change when rank scores were graphically represented (see Figure 2B). Thus, participants who scored best or worst in ROM_max_ at 5°·s^-1^ did not necessarily maintain this position in joint rotations performed at 30 and 60°·s^-1^. On average, participants were ranked 3.1 (5 vs. 30°·s^-1^), 3.2 (5 vs. 60°·s^-1^), and 1.5 (30 vs. 60°·s^-1^) places different within the cohort, equating to 20.4, 21.3, and 9.8% changes in ranking. The maximum changes in rank between tests were 12 (80.0%), 10 (60.0%) and 4 (26.7%), respectively.

Significant strong, positive correlations were found in peak passive joint moments calculated in tests at 5 and 30°·s^-1^ (r*_s_* = 0.67 [0.22 to 0.90], *P* = 0.008), and 60°·s^-1^ (r*_s_* = 0.57 (95% CI, 0.06 to 0.84, *P* = 0.03). However, visual inspection of individual changes revealed no standard pattern of change when rank scores were graphically represented (see Figure 2D). Thus, a participant’s peak joint moment rank at 5°·s^-1^ was not always similar to their rank in tests at 30 and 60°·s^-1^. On average, participants were ranked 2.5 (5 vs. 30°·s^-1^), 2.8 (5 vs. 60°·s^-1^), and 0.9 (30 vs. 60°·s^-1^) places different within the cohort, equating to 16.0, 18.7, and 6.29% changes in ranking. The maximum changes in rank between tests were 9 (60.0%), 9 (60.0%) and 4 (26.7%), respectively.

Significant moderate-to-strong, positive correlations were observed in passive elastic energy measured between tests at 5 and 30°·s^-1^ (r*_s_* = 0.64 (95% CI, 0.18 to 0.87), *P* = 0.01), and 60°·s^-1^ (r*_s_* = 0.59 (95% CI, 0.09 to 0.85, *P* = 0.02). However, visual inspection of individual changes revealed no standard pattern of change when rank scores were graphically represented (see Figure 2E). Thus, a participant’s passive elastic energy rank at 5°·s^-1^ was not necessarily the same as the rank at 30 and 60°·s^-1^. On average, participants were ranked 2.5 (5 vs. 30°·s^-1^), 2.8 (5 vs. 60°·s^-1^), and 0.9 (30 vs. 60°·s^-1^) places different within the cohort, equating to 16.9, 18.7, and 6.2% changes in ranking. The maximum changes in rank between tests were 7 (46.7%), 9 (60.0%) and 4 (26.7%), respectively.

Significant strong, positive correlations were found in MAC stiffness calculated through the last 10° in 5 and 30°·s^-1^ (r*_s_* = 0.60 (95% CI, 0.12 to 0.86, *P* = 0.02), and 60°·s^-1^ (r*_s_* = 0.66 (95% CI, 0.21 to 0.86, *P* = 0.008). However, visual inspection of individual changes revealed no standard pattern of change when rank scores were graphically represented (see Figure 2H). Thus, a participant’s MAC stiffness rank at 5°·s^-1^ was not always similar to their rank in tests at 30 and 60°·s^-1^. On average, participants were ranked 2.7 (5 vs. 30°·s^-1^), 3.1 (5 vs. 60°·s^-1^), and 2.4 (30 vs. 60°·s^-1^) places different within the cohort, equating to 17.8, 20.4, and 16.0% changes in ranking. The maximum changes in rank between tests were 10 (66.7%), 6 (40.0%) and 7 (66.7%), respectively.

Significant strong, positive correlations were found in MAC_0-10_ in tests at 5 and 30°·s^-1^ (r*_s_* = 0.74 (95% CI, 0.4 to 0.9, P = 0.002) and 60°·s^-1^ (r*_s_* = 0.72 (95% CI, 0.3 to 0.9, *P* = 0.003). Visual inspection of individual changes showed no standard pattern of change was observed in rank scores graphically represented (see Figure 2J). On average, participants were ranked 2.3 (5 vs. 30°·s^-1^), 2.1 (5 vs. 60°·s^-1^) and 1.7 (30 vs. 60°·s^-1^) places different within the cohort, equating to 15.1, 14.2, 11.6% changes in ranking. The maximum changes in rank between tests were 7 (46.7%), 9 (60%) and 5 (33.3%), respectively.

Significant moderate-to-strong, positive correlations were observed in MAC_0-20_ in tests at 5 and 30°·s^-1^ (r*_s_* = 0.53 (95% CI, 0.01 to 0.8), *P* = 0.04, respectively), and 60°·s^-1^(r*_s_* = 0.54 (95% CI, 0.3 to 0.8, *P* = 0.04). Visual inspection of individual changes showed no standard pattern of change was observed in rank scores graphically represented (see Figure 2L). On average, participants were ranked 3.1 (5 vs. 30°·s^-1^), 3.2 (5 vs. 60°·s^-1^) and 2.1 (30 vs. 60°·s^-1^) places different within the cohort, equating to 20.4, 21.3, 14.2% changes in ranking. The maximum changes in rank between tests were 10 (66.7%), 10 (66.7%) and 6 (40.0%), respectively.

Significant moderate-to-strong, positive correlations between MAC_0–ROMmax_ in tests at 5 and 30°·s^-1^ (r*_s_* = 0.70 (95% CI, 0.3 to 0.9), *P =* 0.007), and 60°·s^-1^ (r*_s_* = 0.60 (95% CI, 0.1 to 0.8, *P* = 0.02). However, visual inspection of individual changes revealed no standard pattern of change when rank scores were graphically represented (see Figure 2N). On average, participants were ranked 2.8 (5 vs. 30°·s^-1^), 2.9 (5 vs. 60°·s^-1^) and 2.0 (30 vs. 60°·s^-1^) places different within the cohort, equating to 8.9, 9.8, 8.9% changes in ranking. The maximum changes in rank between tests were 7 (46.7%), 8 (53.3%) and 6 (40.0%), respectively.

Overall, these results showed that tests at faster stretching speeds (≥30°·s^-1^) or with muscles voluntarily active provided different (i.e. new) information to slow, passive stretches, as evidenced by the between-test variability in the rank of participants within the cohort. This is important because conclusions made from test outcomes are usually based on an individual’s score relative to a cohort (sample) or population, and a change in an individual’s rank within a cohort or population would affect the conclusions drawn from the test. Thus, to gain insight into an individual’s maximum ROM capacity or tissue stiffness characteristics under varying conditions, tests under each condition appear to be required.

**Figure 2.** ***Left panel (A, C, E, G, I, K, M):*** Relationships between maximum joint range of motion, peak passive joint moment, passive elastic energy, MAC stiffness calculated through the last 10°, from neutral to 10° and 20° of dorsiflexion, and from 0° to ROM_max_ at 5°·s^-1^ (x-axis), and those at 30 and 60°·s^-1^ (y-axis) from Session 2. Overall, moderate-to-strong significant relationships were found between variables from tests performed at 30 or 60 and 5°·s^-1^. ***Right panel (B, D, F, H, J, L, N):*** Individual relative (to the cohort mean, Z scores) change in scores for each dependent variable obtained at each joint rotation velocities. The non-standard changes in scores obtained at each joint rotation velocity possibly suggests that different results can be drawn from these tests.
