# Supplementary material for: Reliability of isokinetic tests of velocity‐ and contraction intensity‐dependent plantar flexor mechanical properties
Source: Scand J Med Sci Sports. 2021 Mar 23;31(5):1009–25. doi: 10.1111/sms.13920 (PMC8251531; doi:10.1111/sms.13920)
Supplement: Supplementary file 8 — Appendix S8 [file SMS-31-1009-s003.docx]

**SUPPLEMENTAL MATERIAL 3**

**Relationship between the number of passive stretching trials and changes in maximal voluntary isometric contraction moment**

The question arises as to whether the performance of more stretch trials by some participants (as they try to achieve their ROM_max_) might impact on muscle force production or MAC stiffness; i.e. whether the first stretch tests then impact on later tests in the battery. To determine whether the number of stretches might impact on changes in muscle force production (used as an indicator of muscle function), associations between the number of stretches and changes in maximal voluntary isometric joint moment production (MVIC) were examined.

As shown in Figure 1 A and B, Pearson’s product-moment correlations showed no associations between the total number of stretching trials (all stretching velocities) and changes in MVIC (Session 1: *r* = -0.35 [-0.73 to 0.19], *P* = 0.196; and Session 2: *r* = -0.35 [-0.73 to 0.19], *P* = 0.196). Thus, no evidence of effect of stretch number was found.

**Fig 1.** Relationships between changes in maximal voluntary contraction before and after Experiment 1 and the number of trials tested in Sessions 1 (A) and 2 (B). Significant relationships between changes in MVC and the number of trials in Sessions 1 and 2 were not observed.
