# Supplementary material for: Reliability of isokinetic tests of velocity‐ and contraction intensity‐dependent plantar flexor mechanical properties
Source: Scand J Med Sci Sports. 2021 Mar 23;31(5):1009–25. doi: 10.1111/sms.13920 (PMC8251531; doi:10.1111/sms.13920)
Supplement: Supplementary file 9 — Appendix S9 [file SMS-31-1009-s005.docx]

**SUPPLEMENTAL MATERIAL 4**

**Effect of *post-hoc* passive trial selection criteria on test-retest reliability (Experiment 1)**

**Background:** Testing passive maximum joint range of motion (ROM_max_) and resistance to stretch (peak passive joint moment) in isokinetic dynamometers requires the performance of several trials to provide confidence that the participant reached the true maximum tolerable range of motion. When performing multiple trials, one important question arises as to which trial to use for analysis, i.e. whether all trials should be averaged, only the best trial accepted, or the researcher or clinician should adopt a specific selection criterion?

This decision is important because different criteria might significantly affect between-day reliability. In Table 1 below, the reliability of passive trials selection was investigated using three criteria. When several trials are performed it is common to average all trials with the intent to narrow the confidence of interval of the variable and therefore give more certainty to the findings. However, this method is sometimes problematic because of variations in ROM_max_ achieved between trials, which compromise the degree of certainty and affect the reliability results.

Another method commonly used is to take the trial with greatest score, here referred as ‘best’. Despite this method possibly indicating the participant’s maximal ability to stretch, it may add random variability due to chance results/outcomes. That is, if that score cannot be repeated with a certain degree of confidence then it may have occurred randomly. Alternatively, the researcher or clinician might adopt a criterion (here referred as ‘criterion adopted’ (CA)). In the present study we developed a criterion that the two trials with least ROM_max_ difference, and the trial with the greatest ROM_max_ if not within them, were inspected for the variability of the joint moment-angle relation. When the greatest ROM_max_ presented abnormal variations in the joint moment-angle relation or it significantly differed from the two trials with least ROM_max_ difference, the best of the two trials with least difference in ROM_max_ was selected for analysis.

Relative between-day (test-retest) reliabilities were calculated for passive ROM_max_ and peak passive joint moment (Experiment 1) for the three criteria for post-hoc trial selection using intra-class correlation coefficients (ICCs) and their respective 95% confidence intervals (CIs). Between-participant relative reliability was calculated using two-way mixed-effect models, absolute agreement (systematic errors) single (non-averaged) scores (ICC_2,1_) for the variables from the best and CA criteria for *post-hoc* trial selection, whereas the ICC values of the average scores were used for the average criterion.

The relative (ICC_2,1_) and absolute (typical error, MDC, and CV%) between- and within-participant test-retest reliability statistics for ROM_max_ and peak passive joint moment using the average, best, and analysed selection of analysis criteria are presented in Table 1. ICC_2,1_ ranged from moderate-to-good for ROM_max_ using the average and CA criteria, and ranged from poor-to-good using the best criterion. Overall, there was an increase in ICC_2,1_ with stretching velocity (5 to 30 to 60°∙s^-1^) for the average and analysed selection of trial analysis criteria, whereas it ICC_2,1_ decreased with stretching velocity for the best criterion. Standard error of measurement (SEM), coefficient of variation (CV), and minimal detectable change (MDC) values consistently differed between stretch velocities for all analysis criteria. However, smaller SEM, CV, and MDC were observed for the CA compared to the average and best criteria for all stretching velocities.

ICC_2,1_ ranged from moderate-to-good for peak passive joint moment for all criteria and were, overall, similar across stretching velocities. Marginal but consistent changes across stretching velocities were observed in SEM, CV, and MDC values for average criterion and the CA. However, marginally smaller SEM, CV, and MDC were observed for the best compared to the average criterion and the CA at the slower stretching velocity, yet larger values were observed at faster stretching velocities for the best selection of trial analysis criterion.

Based on these results, we recommend the use of CA when smaller between-day absolute (SEM and CV) reliability is necessary and/or significant variations in ROM_max_ between trials are observed that affects calculations of other variables that rely on higher reliability of the testing measure (e.g. passive peak joint moment or musculoarticular stiffness). Alternatively, the average method might be used when consistent change in rank between days consistently is need, i.e. between-day relative reliability as indicated by ICC.

| **Table 1.** Summary of relative and absolute (test-retest) reliability results for different analysis methods. Intra-class coefficient (ICC_2,1_) indicates relative reliability whilst standard error of measurement (SEM, i.e. typical error), coefficient of variation, and minimal detectable change (MDC) indicate absolute reliability. “Average” indicates the average of all trials performed in each velocity; “best” indicates the greatest (best) score of all trials; “CA”, criterion adopted, indicates the *post-hoc* criterion adopted in the present study (see Methods for further detail). | | | | | | | | | | | | | | | |
| --- | --- | --- | --- | --- | --- | --- | --- | --- | --- | --- | --- | --- | --- | --- | --- |
|  |  | 5°^.^s^-1^ | | | |  | 30°^.^s^-1^ | | | |  | 60°^.^s^-1^ | | | |
|  |  | ICC | SEM | CV | MDC |  | ICC | SEM | CV | MDC |  | ICC | SEM | CV | MDC |
|  | Criterion of analyses |  |  |  |  |  |  |  |  |  |  |  |  |  |  |
| Maximum dorsiflexion angle (°) | Average | 0.62 (-0.1 to 0.87) | 4.7 | 11.4 | 9.2 |  | 0.84 (0.54 to 0.94) | 2.3 | 4.5 | 4.4 |  | 0.77 (0.35 to 0.92) | 2.7 | 5.2 | 5.3 |
|  | Best | 0.54 (0.10 to 0.82) | 4.3 | 10.0 | 8.4 |  | 0.30 (-0.20 to 0.69) | 2.5 | 4.4 | 4.9 |  | 0.16 (-0.37 to 0.6) | 2.5 | 4.3 | 4.9 |
|  | CA | 0.54 (0.08 to 0.82) | 4.2 | 10.0 | 8.2 |  | 0.79 (0.46 to 0.93) | 1.8 | 3.7 | 3.6 |  | 0.87 (0.48 to 0.96) | 1.3 | 3.3 | 2.5 |
| Peak passive joint moment (Nm) | Average | 0.82 (0.47 to 0.94) | 32.0 | 17.6 | 62.7 |  | 0.84 (0.54 to 0.95) | 33.5 | 12.5 | 65.6 |  | 0.86 (0.59 to 0.95) | 39.2 | 11.6 | 76.8 |
|  | Best | 0.80 (0.50 to 0.93) | 27.5 | 15.6 | 53.8 |  | 0.63 (0.20 to 0.86) | 38.7 | 12.1 | 75.6 |  | 0.67 (0.25 to 0.88) | 46.0 | 13.2 | 90.2 |
|  | CA | 0.68 (0.27 to 0.88) | 34.6 | 17.0 | 67.8 |  | 0.75 (0.41 to 0.91) | 34.7 | 12.2 | 68.0 |  | 0.82 (0.55 to 0.94) | 35.8 | 9.8 | 70.1 |
|  | | | | | | | | | | | | | | | |
